# Remediation of *ABCG5*-Linked Macrothrombocytopenia With Ezetimibe Therapy

**DOI:** 10.3389/fgene.2021.769699

**Published:** 2021-11-22

**Authors:** Libin Deng, Jingsong Xu, Wei Chen, Shicheng Guo, Robert D. Steiner, Qi Chen, Zhujun Cheng, Yanmei Xu, Bei Yao, Xiaoyan Li, Xiaozhong Wang, Keyu Deng, Steven J. Schrodi, Dake Zhang, Hongbo Xin

**Affiliations:** ^1^ The Second Affiliated Hospital of Nanchang University, Nanchang, China; ^2^ Institute of Translational Medicine, Nanchang University, Nanchang, China; ^3^ Jiangxi Provincial Key Laboratory of Preventive Medicine, School of Public Health, Nanchang University, Nanchang, China; ^4^ Key Laboratory of Biomechanics and Mechanobiology, Ministry of Education, Beijing Advanced Innovation Center for Biomedical Engineering, School of Biological Science and Medical Engineering, Beihang University, Beijing, China; ^5^ Department of Medical Genetics, University of Wisconsin-Madison, Madison, WI, United States; ^6^ Department of Pediatrics, University of Wisconsin-Madison, Madison, WI, United States; ^7^ Department of Clinical Laboratory, Peking University Third Hospital, Beijing, China; ^8^ Beijing Institute of Heart, Lung & Blood Vessel Disease, Beijing Anzhen Hospital, Capital Medical University, Beijing, China

**Keywords:** platelet, blood, sitosterolemia, hypercholesterolemia, ezetimibe (EZE)

## Abstract

To investigate refractory hypercholesterolemia, a female patient and relatives were subjected to whole-genome sequencing. The proband was found to have compound heterozygous substitutions p. Arg446Gln and c.1118+3G>T in *ABCG5*, one of two genes causing sitosterolemia. When tracing these variants in the full pedigree, all maternally related heterozygotes for the intronic *ABCG5* variant exhibited large platelets (over 30 fl), which segregated in an autosomal dominant manner, consistent with macrothrombocytopenia, or large platelet syndrome which may be associated with a bleeding tendency. *In vitro* cell-line and *in vivo* rat model experiments supported a pathogenic role for the variant and the macrothrombocytopenia was recapitulated in heterozygous rats and human cell lines exhibiting that single variant. Ezetimibe treatment successfully ameliorated all the symptoms of the proband with sitosterolemia and resolved the macrothrombocytopenia of the treated heterozygote relatives. Subsequently, in follow up these observations, platelet size, and size distribution were measured in 1,180 individuals; 30 were found to be clinically abnormal, three of which carried a single known pathogenic *ABCG5* variant (p.Arg446Ter) and two individuals carried novel *ABCG5* variants of uncertain significance. In this study, we discovered that identification of large platelets and therefore a possible macrothrombocytopenia diagnosis could easily be inadvertently missed in clinical practice due to variable instrument settings. These findings suggest that *ABCG5* heterozygosity may cause macrothrombocytopenia, that Ezetimibe treatment may resolve macrothrombocytopenia in such individuals, and that increased attention to platelet size on complete blood counts can aid in the identification of candidates for *ABCG5* genetic testing who might benefit from Ezetimibe treatment.

## Background

Plasma cholesterol homeostasis is maintained by the synthesis, intestinal absorption, and biliary and fecal excretion of sterols. The biosynthesis of cholesterol is a well-defined energy-consuming and feedback-regulated process (Howe et al., 2016). ATP-binding cassette transporters G5 (*ABCG5* gene) and G8 (*ABCG8* gene) usually form a heterodimer (G5G8) which inhibits the absorption of cholesterol and plant sterols by promoting the efflux of these sterols from enterocytes back into the gut lumen, and the secretions from hepatocytes into bile (Wang et al., 2015; Lee et al., 2016; Sun et al., 2021). In contrast, sterol transporter Niemann–PickC1-Like1 (NPC1L1) promotes intestinal cholesterol absorption and biliary cholesterol re-absorption (Jia et al., 2011). Together, they maintain sterol balance without direct involvement in sterol synthesis.

Homozygous or compound heterozygous variants in either *ABCG5* or *ABCG8* cause autosomal recessive sitosterolemia (OMIM 210250) (Myrie et al., 1993; Berge et al., 2000), characterized by elevated plasma levels of plant sterols (Bhattacharyya and Connor, 1974; Myrie et al., 2012; Brautbar et al., 2015). However, patients with *ABCG5/8* pathogenic variants show significant phenotypic heterogeneity (Wang et al., 2004). The other features also variably include hypercholesterolemia, xanthomas, xanthelasma, and premature atherosclerosis. Affected individuals may also suffer from diverse hematological alterations such as a decreased platelet count, an increased mean platelet volume (large platelets/macrothrombocytosis or macrothrombocytopenia if accompanied by reduced platelet count), and/or hemolytic anemia.

Management of sitosterolemia aims to reduce plasma plant sterol accumulation. Ezetimibe, a small molecule inhibitor of NPC1L1 approved for the treatment of hypercholesterolemia (Suchy et al., 2011), is considered the preferred treatment for sitosterolemia (Salen et al., 2004; Yu et al., 2005; Salen et al., 2006; Lutjohann et al., 2008; Tsubakio-Yamamoto et al., 2010; Altemus et al., 2014; Erkan, 2014; Hu and Tomlinson, 2014; Othman et al., 2015; Yoo, 2016). Studies using knock-out mouse models have successfully shown that Ezetimibe treatment can correct multiple symptoms, including reduction of large platelets and restoration of the platelet count to some extent, caused by pathogenic variants in *ABCG5/8*; similarly, clinical trials also show improvement in patients with sitosterolemia after Ezetimibe therapy (Salen et al., 2004; Lutjohann et al., 2008; Tsubakio-Yamamoto et al., 2010; Hu and Tomlinson, 2014; Othman et al., 2015). Nevertheless, the clinical improvement in these patients may be attributed at least in part to plasma cholesterol reduction, and the benefits of reduction of plant sterol storage remain unclear (Ajagbe et al., 2015).

Here we described the identification of two variants of *ABCG5* in a proband with apparent autosomal recessive hypercholesterolemia recalcitrant to statin therapy who exhibited large platelets without dysfunction of blood coagulation, neither platelet counts nor their functions. Unexpectedly, large platelets (over 30 fl) were a dominant phenotype in family members heterozygous for the c.1118+3G>T variant in *ABCG5.* Large platelet syndrome describes a group of unique disorders characterized by the presence of abnormally large platelets and is usually accompanied by thrombocytopenia. Thus, it is also termed macrothrombocytopenia. Functional studies showed that variant can cause deletion of exon 8 in human *ABCG5*, and disruptions of *ABCG5*/*ABCG8* genes recapitulated the occurrence of hypercholesterolemia and large platelets in rats. These variants were ultimately classified as pathogenic and likely pathogenic. Interestingly, the macrothrombocytosis was also successfully treated with Ezetimibe in heterozygotes in the pedigree. Large platelets can be occasionally seen in individuals without other obvious abnormal hematologic findings, and we further sequenced *ABCG5* in a large unrelated cohort of individuals with large platelets to see if *ABCG5* variant heterozygosity is a common cause; this study was carried out to determine whether a subset of individuals with large platelets might be expected to respond to Ezetimibe treatment, as an example of the potential application of pharmacogenomics that benefits from genetic analysis (Yu and Hu, 2021).

## Methods

### Proband Description and Blood Sample Collection

**FIGURE 1:**
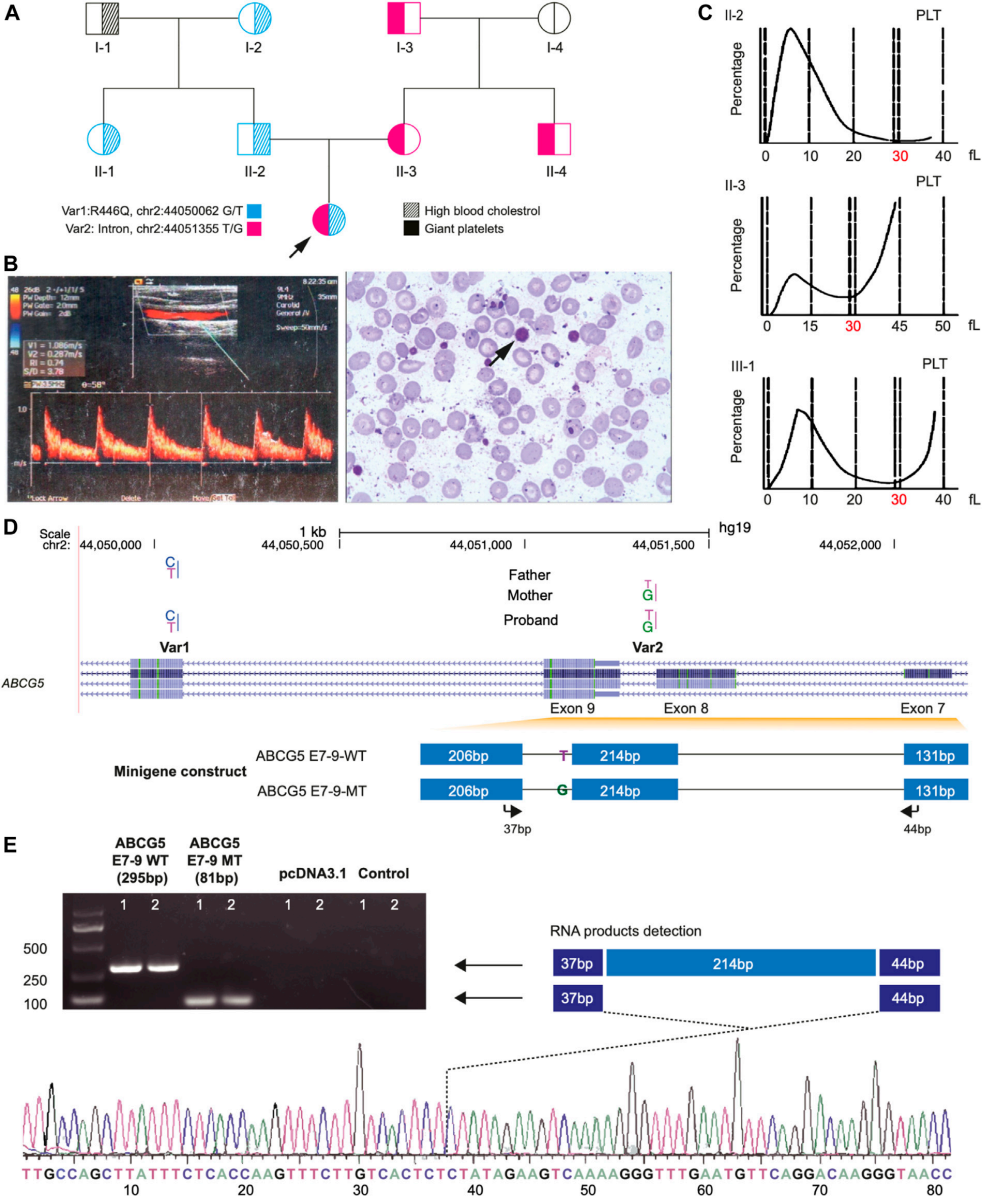
Clinical Phenotypes and Identification of Two Novel Mutations of *ABCG5* Gene of the proband in a pedigree family. Panel **(A)** shows the large platelet in the blood smear **(left)** and the atherosclerotic plaque by B-mode ultrasound measurement from the proband. Panel **(B)**, a pedigree chart shows the phenotype distribution in all family members and the transmission of two novel variants (Var 1 and Var 2) of *ABCG5* in the family. Panel **(C)** shows the size distribution of platelets (PLT) in the proband and her parents. The dash lines represent the platelets’ volume, and the double dash lines indicate 30 fl, in which 40 fl is the up limitation for the normal distribution of PLT. Panel **(D)** shows the *ABCG5* gene model from the NCBI database and the inserted fragments in the constructs for wild type and Var2 mutant in the splicing pattern assay. The arrows indicate the primer pairs used for the detection of the mRNA products which were transcribed from the constructs. Panel **(E)** shows the PCR amplification results of two products generated from wild-type and Var2 mutant constructs, respectively. The product from the wild-type construct is 295 bp, and the product for the mutant is 81 bp due to deleting exon 8 of the *ABCG5* gene. The truncated transcripts were further verified by Sanger sequencing.

A 6-year-old girl with refractory hypercholesterolemia was referred to our institution for the development of a suitable therapeutic regimen. Before the study, atorvastatin had been administered to the patient for more than 2 years, but her plasma total cholesterol level was decreased to only ∼200 mg/dl and large platelets persisted. Additionally, the patient appeared to have atherosclerotic plaque (6 mm × 7 mm) in her left common carotid artery (Figure 1A, right) upon referral. The patient’s initial presenting clinical and laboratory features included profoundly increased levels of total cholesterol (638 mg/dl) and low-density lipoprotein cholesterol (LDL-C, 527 mg/dl); a reduced level of high-density lipoprotein cholesterol (HDL-C, 46 mg/dl) and a slight elevation of plasma β-sitosterol (3.26 mg/dl). Although there was an elevation of β-sitosterol, criteria for the diagnosis of sitosterolemia were initially felt not to be met (Salen et al., 1985). In addition, the patient had abnormally large platelets (Figure 1A, left); over 41% of her platelets had a size over 30 fl. Clinically, Bernard-Soulier syndrome, of which giant platelets are a feature, was excluded as a plausible diagnosis through examination of clotting time and platelet aggregation, which were all normal in the proband (Table 1).

**TABLE 1:** Clinical characteristics of the family members.

	Id									Normal value of rang
	I-1	I-2	I-3	I-4	II-1	II-2	II-3	II-4	III-1	
Gender	Male	Female	Male	Female	Female	Male	Female	Male	Female	
Age	69	66	58	52	45	40	34	32	9	
TC	169.76	213.46	155.07	200.31	212.69	258.7	134.96	97.84	638.06	<200 mg/dl
LDL-C	99	129.93	96.29	123.74	124.9	163.19	71.54	50.66	527.46	≤120 mg/dl
HDL-C	49.88	61.49	43.7	51.43	68.83	67.29	53.75	39.06	45.63	50–60 mg/dl
β-Sitosterol	1.06	1.44	1.58	1.14	1.3	1.23	1.52	1.49	3.26	0.31∼0.80 mg/dl
PLT	275	324	172	298	363	240	250	217	289	85-303 10^9/L
PDW	10.5	10.9	NaN	15.4	11.4	12.1	NaN	NaN	15.7	11∼26.5 fl
MPV	10.2	10.3	NaN	12	10.6	10.8	NaN	NaN	12.1	7.6∼13.2 fl
P-LCR	25	26.1	NaN	39.2	28.5	30.1	NaN	NaN	41	13∼43%
PT			11 (9–13)				9.4 (9–13)	10 (9–13)	10 (9–13)	
APTT			29 (20–40)				26.8 (20–40)	28 (20–40)	27.3 (20–40)	
TT			17.1 (14–24)				15 (14–24)	16.7 (14–24)	15 (14–24)	
FIB			3.1 (2–4)				3.84 (2–4)	3.62 (2-4)	3.6 (2–4)	
PTA			120 (70–150)				106.8 (70–150)	117 (70–150)	116.5 (70–150)	
PAgT			61 (35–75)				65.7 (35–75)	63 (35–75)	64.3 (35–75)	

Total cholesterol, TC, Low-density lipoprotein cholesterol; LDL-C, High-density lipoprotein cholesterol; HDL-C, Platelet count; PLT, Platelet distribution width; PDW, Mean platelet volume; MPV, Platelet-large cell ratio; P-LCR, prothrombin time; PT, activated partial thromboplastin time; APTT, thrombin time; TT, fibrinogen coagulative time; FIB, prothrombin time activity; PTA, platelet agglutination test, PAgT.

To track genetic variants in the full pedigree, we also collected blood samples from maternal and paternal relatives (I-1,2,3,4, and II-1,4). DNA was extracted from 400 μl whole blood per sample using the (Qiagen, Hilden, Germany), and all blood samples were stored at −80°C before usage. For screening for candidate *ABCG5* variants in a general population, we also enrolled individuals over 2 months in 2019, the Second Affiliated Hospital of Nanchang University, Beijing Anzhen Hospital of Capital Medical University, and Peking University Third Hospital. The samples were examined in the Second Affiliated Hospital of Nanchang University. In all, 30 patients had abnormal platelet size distribution and underwent *ABCG5* gene sequencing.

### Gene Panel Test for Familial Hypercholesterolemia

We designed a custom panel of DNA oligonucleotide primers covering 216 amplicons using the Ion AmpliSeqTM platform (Supplementary Table S1, Life Technology, Thermo Fisher, USA). These amplicons covered all coding exons of four familial hypercholesterolemia (FH) causal genes (*APOB*, *LDLRAP1*, *LDLR*, and *PCSK9*). Sequencing Libraries were barcoded (IonXpress Barcode Kit, Life Technologies) and equalized (Ion Library Equalizer Kit) to a final concentration of approximately 100 pM. Emulsion PCR was performed using the OneTouch DL instrument, and template-positive Ion Sphere particles were enriched using the OneTouch ES instrument according to the manufacturer’s instructions. Sequencing was performed on a 318 chip on the Ion Torrent PGM following the recommended protocol. Reads were aligned to hg19 and variants were called using the TorrentSuite (version 4.0.2).

### Whole Genome Sequencing Analysis and Identification of Pathogenic Variants

WGS was conducted in the proband and her parents. Each sequencing library with an average insert size of 250 bp was loaded into an Illumina HiSeq X ten. Roughly 90 Gb of high-quality data in 150 bp pair-end reads was obtained for each library, reaching an average of 30-fold genome coverage for each individual. The quality evaluation was performed using FastQC software, and sequences from adapters or having low Q-score were removed with cutadapt software (Martin, 2011). The sequencing reads were mapped to the reference human genome (hg19) by the BWA algorithm (Li and Durbin, 2009). PCR duplicates were removed with Picard software. Processed bam files of humans were processed via local indel realignment and base-quality recalibration using the Genome Analysis Tool Kit (GATK) (McKenna et al., 2010). Subsequently, sorted BAM files were used for SNV calling and the UnifiedGenotyper method based on a Bayesian genotype likelihood model was applied with GATK. All detected SNVs were annotated using ANNOVAR. VarSelect in TGex platform was applied to filter for variants in WGS data of the family trio, to identify variants most likely responsible for the phenotypes of the proband (Dahary et al., 2019).

### Blood Test and Analysis of Plasma Sterol Levels

The blood lipid measurements were routinely performed by the second Affiliated Hospital of Nanchang University. β-Sitosterol and other plant sterols were analyzed by liquid chromatography-mass spectrometry (AB SCIEX Triple Quad 4500) according to previous studies(Kasama et al., 1987; Hidaka et al., 1990). For each sample, a volume of 20 μl was injected into Agilent Eclipse Plus reversed-phase column (C18, 2.1 × 50 mm). The column temperature was maintained at 35°C. The mobile phase was acetonitrile-methanol (4:1, v/v) at a flow rate of 0.6 ml/min. The mass spectrometer was operated in positive ion polarity mode in the extended dynamic range (1,700 m/z, 2 GHz) with the following parameters: Curtain Gas (GUR) 40 psi; Collision Gas (CAD) 9; IonSpray Voltage (IS) 5,500.0 V; Temperature (TEM) 350°C; Ion Source Gas 1 (GS1) 50 psi; NC 3V. Blood samples from 10 healthy individuals were examined as control samples.

### Platelet Size Analysis

Two ml of peripheral blood was collected using standardized tubes (INSEPACK ST serials, Beijing, China) and all tubes were stored at room temperature. Within 30 min, these samples were analyzed on SysmexXE-2100 Haematology System (Sysmex Corporation, Kobe, Japan) for lipid profile and coagulation tests following the methods previously developed protocols (Table 1) (Barsam et al., 2011; Depoorter et al., 2015). Large platelets are identified based on the volume (>30 fl). For each sample, we prepared two blood films dyed by Wright’s dye for platelet morphology analysis using a BX53 microscope (OLYMPUS, Japan). The criteria for large platelets is a diameter greater than 4 microns. All blood films were reviewed by two independent examiners.

### Splicing Pattern Assay for ABCG5 Var2

Var2 is located at a splice region of *ABCG5*, and we performed the splicing pattern assay to verify its functional effect. Genomic DNAs including reference (WT) or *ABCG5* variant were isolated from peripheral whole blood of all individuals in the study with Blood Genomic DNA Mini Kit (CWBIO, CW 2087S) and PCR was performed using PrimeSTAR® MaxDNA Polymerase (TAKARA, R045A). The 947 bp PCR fragments of *ABCG5* gene, spanning from part of exon 7 (44 bp) to part of exon 9 (37 bp) with exon8 (214 bp) and its flanking intron 7 and 8, were amplified by the primer pair, *ABCG5*-minigene_Forward (5′-GCG​GTA​CCG​CGG​AAA​TGC​TTG​ATT​TCT​T-3′) with KpnI restriction site and *ABCG5*-minigene_Reverse (5′-GCCTCGAG TTA​AAG​GAG​GAA​CAA​ACC​CAT​GA-3′) with XhoI restriction site. The genomic fragments (both WT and variant, MT) containing the intron of interest were cloned into the pcDNATM-3.1 (+) vector and then all cloned plasmids were verified by sequencing to confirm whether the insertion contained the WT or MT.

HepG2 cells were cultured in DMEM (Gibco) supplemented with 10% FBS (Gibco). After seeded in 6-well plates for 24 h, HepG2 cells were transiently transfected with 1ug prepared vectors with the allele of the WT or MT and corresponding empty vectors respectively, using Superfectin II *In Vitro* siRNA Transfection Reagent (Shanghai Pufei Biotech). After 48 h, total RNA was extracted by TRIzol reagent (Invitrogen) and 1 mg total RNA was used for reverse transcription using PrimeScript RT reagent kit with gDNA eraser (Takara) according to the manufacturer’s instructions. The primer sequences of quantitative RT-PCR were: *ABCG5*-MGQ_Forward (5′-CGG​TTA​CCC​TTG​TCC​TGA​AC-3′) located in exon 7 and *ABCG5*-MGQ_Reverse (5′-TGC​CAG​CTT​ATT​TCT​CAC​CA-3′) located in exon 9. Quantitative RT-PCR was performed with SYBR Green dye using ViiA7Real-Time PCR System (Applied Biosystems). The relative mRNA expression was calculated by the comparative Ct method using *GAPDH* as a control. PCR reactions were performed in triplicate. The RT-PCR products were separated on a 2% agarose gel and detected with Chemidoc Xrs Gel Doc Xr (Bio-Rad Universal Hood Ii 2, USA).

### Generation of ABCG5/ABCG8 Double Knockout Rat

*ABCG5/ABCG8* double knockout rat model was created by Beijing Biocytogen. In brief, a 19 Kb region was knocked out using a CRISPR/Cas9 system, with two sgRNAs targeting one site in the intron 4 of *ABCG5*, and the other in the intron 6 of *ABCG8*. The design was adapted from the protocol developed by Yu L, et al. The sgRNA activity was evaluated by the UCA^TM^ (Universal CRISPR Activity Assay), developed by Biocytogen. By zygote microinjection, transferred zygotes of SD rats were obtained and the founders were positively confirmed by PCR product sequencing. The genotyping primers were Forward: 5′-cta​ggt​cca​cca​agc​cat​gtg​aac​a and Reverse: 5′-att​ttc​tgg​gca​ccc​tgt​gtt​cca​c. The animal study was approved by the Ethics Committee of the Second Affiliated Hospital of Nanchang University (SYXK-20150001).

### Sanger Sequencing of ABCG5

Exon amplification was performed using LA Taq (Takara, Osaka, Japan). Supplementary Table S2 listed all the primers for amplification of 13 exons of *ABCG5* extending 50 bp towards both upstream and downstream to cover the splice regions and corresponding PCR protocols for each primer pair. All reactions were performed on a PTC-200 Peltier Thermal Cycler (MJ Research, MA, USA). PCR products were sequenced by Sangon Biotech (Shanghai, China), and the results were manually checked using SeqMan (DNAstar 5.0, WI, USA).

### Statistical Analysis

The *t*-test was used to compare the mean difference of the blood lipid levels in the rat model using R 3.4(R Core Development Team, 2018).

## Results

### Identification of Compound Heterozygous Variants in ABCG5

This proband had hypercholesterolemia with atypical changes in the β-Sitosterol level in comparison with previous reports (Supplementary Table S3). To identify pathogenic variants responsible for the hypercholesterolemia of the proband, a customized Gene Panel Test was initially performed to screen for variants within all coding regions of four common familial hypercholesterolemia genes: *APOB*, *LDLR*, *LDLRAP1*, and *PCSK9*. The sequencing results showed that no family member carried any rare coding variants (<1%, MAF in CHB of 1,000 genomes) in these genes. Next, we performed WGS for the proband and her parents (∼30X coverage on average for each person). Two heterozygous variants of the *ABCG5* gene were found in the proband, and no other variant was observed in the known genes related to hypercholesterolemia. The proband inherited Var1 (chr2:44050062G/T, NM_022436.3:c.1337G>A, p. Arg446Gln) from her father and Var2 (chr2:44051355T/G, splice region, c.1118+3G>T) from her mother. These two variants (Var1 and Var2) were further verified by Sanger sequencing (Supplementary Figure S1).

Var1 was reported to be a variant of uncertain significance for sitosterolemia in both Clinvar (Accession: VCV000289811) and gnomAD (2-44050062-C-T, allele frequency: 6.74e-5 and no homozygote). While at the same position, p. Arg446Ter (Accession: VCV000030485), has been previously reported as pathogenic for sitosterolemia and hypercholesterolemia (Wang et al., 2014; Buonuomo et al., 2017; Pek et al., 2018). Var2 was not documented in either Clinvar or gnomAD; it was predicted to be a splice site variant. The blood total cholesterol level of the proband was similar to the level in previously reported individuals with biallelic *ABCG5* variants, ranging from 116 mg/dl to 870 mg/dl (Supplementary Table S3).

Subsequently, these two variants were examined in all family members. The results showed that these variants were transmitted across all three generations in heterozygous form (Figure 1B). Var1 was specific to the paternal side, and Var2 was only detected in the maternal lineage. However, individual I-1 did not carry any variants in the *ABCG5* gene. In addition, all maternal members heterozygous for Var2 exhibited the large platelet phenotype without dysfunction of blood coagulation (Figure 1C; Table 1; Supplementary Figure S2).

### Functional Assessment of Var2 Mutation

Since Var2 was predicted to be a splice site variant, we examined if Var2 can result in novel splicing transcripts of *ABCG5* (Figure 1D). As shown in Figure 1E, the mRNA transcripts from the reference and Var2 fragments were 295 bp and less than 100 bp, respectively. Subsequent Sanger sequencing verified that this “truncated” transcript was 81 bp and excluded exon 8. Therefore, this result suggests that Var2 leads to deletion of exon 8 of *ABCG5* during transcription, and the creation of a premature termination codon triggering nonsense-mediated mRNA decay.

### Treatment With Ezetimibe

**FIGURE 2:**
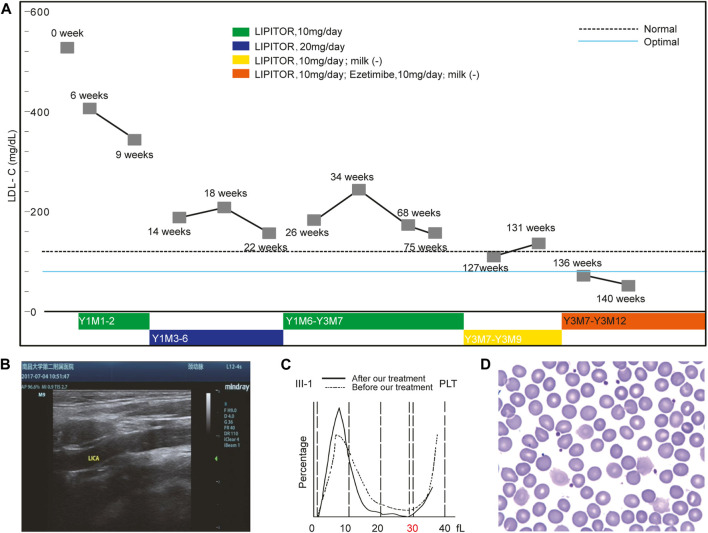
Ezetimibe Corrected the Phenotypes of the Proband. Panel **(A)** shows the changes in the LDL-C levels under an optimized treatment regimen. Panel **(B)** shows the disappearance of the aforementioned atherosclerotic plaque revealed by the ultrasound scan after Ezetimibe treatment. Panel **(C)** shows the comparison of platelet size distributions before and after Ezetimibe treatment in the proband. Panel **(D)** shows the disappearance of the large platelet in the blood smear of the proband after treatment of Ezetimibe.

Before the study, atorvastatin 10 mg/day was first administered to the proband and her plasma LDL-C level gradually decreased to around 200 mg/dl within 2 years (Figure 2A). Although the proband’s serum LDL-C level was reduced to about 150 mg/dl when atorvastatin was increased to 20 mg/day, she experienced severe adverse effects (Supplementary Table S4). By reducing atorvastatin back to 10 mg/day, the proband’s LDL-C level remained around 200 mg/dl.

Considering the insufficient response to atorvastatin which aims to inhibit cholesterol biosynthesis, we suspected that the proband may have an abnormality in cholesterol excretion instead. At that point, the treating physician suggested stopping cow milk intake (450 ml/day), following a similar infant case with a limited increase in β-Sitosterol where breastfeeding was arrested to ameliorate the symptoms (Rios et al., 2010).

After 1 month, the proband’s LDL-C level was reduced to 130 mg/dl without further decrease. The effect caused by the reduction of cholesterol intake supported the hypothesis of a cholesterol excretion dysfunction since this strategy is not effective for individuals with abnormal cholesterol synthesis. WGS analysis identified compound heterozygous variants in *ABCG5* responsible for abnormal cholesterol excretion consistent with the known disorder sitosterolemia, which supported our hypothesis. Following this finding, the physician in our group recommended a combination of atorvastatin (10 mg/day) and Ezetimibe (10 mg/day) be administered to the proband.

Within 2 months, the patient’s LDL-C level was further decreased to an optimal level (<80 mg/dl). After treatment for 39 months, atorvastatin was discontinued but Ezetimibe treatment was continued with no milk intake. Thus far, the patient’s LDL-C level has been maintained at around 80 mg/dl without any obvious adverse effects. An ultrasound scan 2 months following the revised treatment showed that the proband’s atherosclerotic plaque disappeared from her left carotid artery (Figure 2B).

Interestingly, the proband’s macrothrombocytopenia nearly completely resolved within 1 month after the addition of Ezetimibe while the β-Sitosterol level was reduced to normal values (0.83 mg/dl). The size distribution of platelets normalized, and only 25% of platelets remained over 30 fl (Figures 2C,D). ABCG5 functional inactivation leads to relatively active NIPIC, targeting the NIPIC by Ezetimibe was shown to restore the balance of cholesterol absorption and excretion maintained by them. These results suggested that the macrothrombocytopenia of the proband was likely caused by the *ABCG5* variant. More unexpectedly, all Var2 heterozygotes in the family had large platelets, and after 1 month of Ezetimibe treatment alone, the macrothrombocytopenia of her maternal uncle (II-2, Figure 2) also disappeared (Supplementary Figure S3).

Disruptions of the ABCG5/ABCG8 genes mimic phenotypes of the proband in double knockout rat.

**FIGURE 3:**
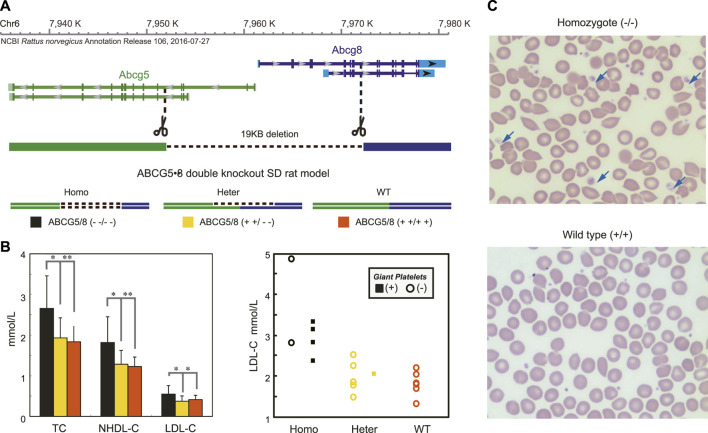
Disruptions of *ABCG5/8* Genes Mimicked the Phenotypes of the Proband in the Double Knockout Rats. Panel **(A)** shows the strategy for the generation of *ABCG5/8* double knockout using the CAS9/sgRNA system. Panel **(B)** showed significantly increased blood lipid levels **(left)** and the large platelet **(right)** in the homozygotes compared with two other groups. For each group, the blood lipid levels and the large platelet were determined from randomly selecting rats. The values of the blood lipid levels are expressed as the mean ± SE. *n* = 6. *p < 0.05, **p < 0.01. The large platelet was detected in over 80% of homozygotes, and none was found in the wild-type strain. Panel **(C)** shows a blood smear for three groups of the rat model.

To further determine if heterozygotes of *ABCG5/ABCG8* genes could drive hypercholesterolemia and macrothrombocytopenia phenotypes, we created a double knockout rat (Figure 3A), having a growth curve similar to the WT strain without visible abnormalities (Supplementary Figure S4A) and succesful depletion of ABCG5 and ABCG8 expression in liver and intestine (Supplementary Figure S4B). As expected, the G5G8^--/--^ rats had a significant elevation of blood lipid levels (Figure 3B, left) with large platelets (4 out of 6 animals) compared with wild-type rats. However, the alterations in blood lipids were not strictly correlated with the occurrence of large platelets (Figure 3B, right), further supporting the observations of phenotypic heterogeneity of *ABCG5/ABCG8* variants. In particular, one heterozygous rat (G5G8^+ +/- -^) also had this biased distribution of platelet size (Figure 3C), similar to the maternal kindred with Var2, suggesting that heterozygotes of this *ABCG5* variant might be sufficient to cause macrothrombocytopenia. Considering these functional studies, Var2 may be classified as “Likely pathogenic” according to the standards and guidelines of the American College of Medical Genetics and Genomics (ACMG) (Richards et al., 2015): in this case PS3, our functional studies show a deleterious effect; PM2, absent in population databases. Addtionally, the homozygous KO rats had significantly higher β-Sitosterol level (mean, 7 mg/dl) than WT strain (mean, 2.5 mg/dl) when even being fed by normal diet. We further treated 2 KO rats with Ezetimibe 0.2mg/Kg. Their serum TC reduced from 2.3 and 2.4 mmol/L to 1.7 and 1.9 mmol/L , and β-Sitosterol level reduced from 6.5 and 7.5 mg/dl to 2.6 and 2.7 mg/dl.

### Screening for ABCG5 Variant Carriers in the General Population

We decided to determine whether *ABCG5* variants are associated with large platelets in the general population. Routine automated blood cell counting systems differentiate blood cells by their size and do not recognize large platelets as platelets, these instruments may not accurately detect macrothrombocytopenia. The mean platelet volume also does not reflect actual platelet size in the case of large platelets. Platelet count should therefore be determined manually in a calculating chamber or on peripheral blood smears when suspecting the condition. On routine clinical laboratory examination, biased platelet distribution can be observed, with an abnormal “NaN” value instead of specific values. Without other indicators, large platelets may be easily overlooked.

**FIGURE 4:**
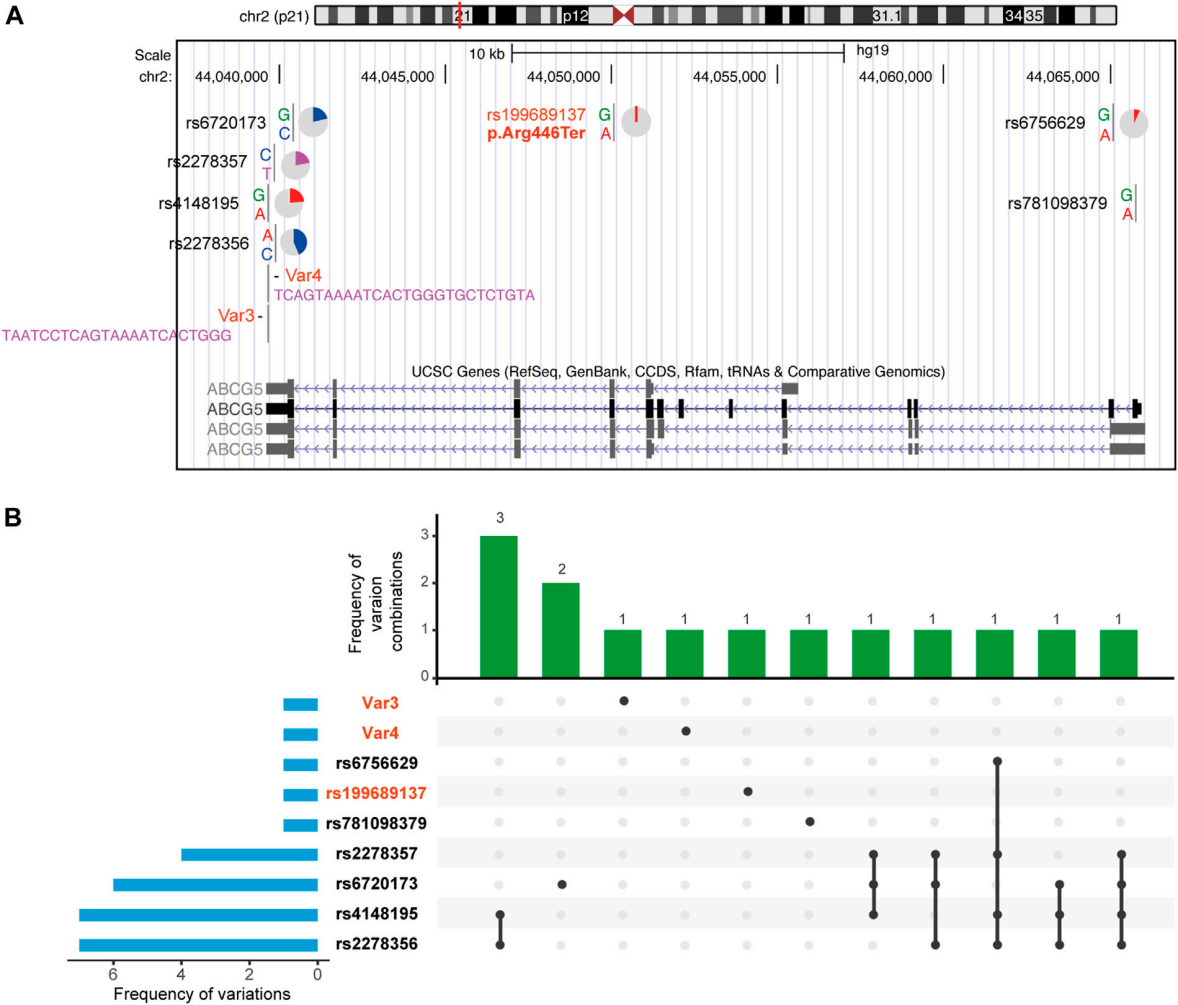
Nine ABCG5 variants in 30 individuals with abnormal platelet size distribution. Details of each variation are in Supplementary Table S4. Genetic variations of them are more commonly seen in the 3′UTR of the gene model. Except for 3 pathogenic and likely pathogenic variations are heterozygotes in affected individuals, other variations are all homozygotes.

Here, we intended to examine if platelet size could be used to identify *ABCG5* heterozygotes (Methods). We collected platelet size distribution results for all individuals having this examination within 2 months in several hospitals. Among 1,180 individuals screened, 30 had evidence of large platelets (>30 fl, Supplementary Figure S5). In *ABCG5* sequencing of the 30 individuals (Figure 4, Supplementary Table S5), we identified a known pathogenic variant (p.Arg446Ter, gnomad:2-44050063-G-A; allele frequency:1.70e-4 and no homozygotes) in one individual. In addition, two novel variants, Var3 (hg19-chr2:44039644, NM_022436:c.*610_*611insCCCAGTGATTTTACTGAGGATTA) and Var4 (hg19-chr2:44039650, NM_022436:c.*604_*605insTACAGAGCACCCAGTGATTTTACTGA,hg19), insertions in 3’ UTR region, were also identified in three other individuals, respectively. These are classified as variants of uncertain significance (no record in gnomAD, thus only having PM2 evidence according to ACMG standards and guidelines). Hence, this population-based work along with segregation of an *ABCG5* variant in a large pedigree with sitosterolemia identifies heterozygosity for specific *ABCG5* variants as an additional genetic cause of macrothrombocytopenia. Our data suggest that 2.5% of individuals requiring complete blood counts may have large platelets, and roughly 10% of those with large platelets may have *ABCG5* variants that are causative. Therefore, screening for large platelets may be helpful to identify heterozygotes for *ABCG5* pathogenic variants; conversely, sequencing *ABCG5* in individuals with large platelets may be useful in identifying patients amenable to Ezetimibe treatment, if it were shown to be effective and beneficial in future large clinical trials.

## Discussion

We identified a proband with profound hypercholesterolemia, recalcitrant to atorvastatin therapy. The pedigree analysis did not support dominant inheritance and gene panel testing for several genes associated with FH was non-diagnostic. *ABCG5* Var1 did not behave in a classical dominant way that most other variants responsible for FH lead to abnormal lipid levels of affected individuals when they were young. It is important to note that the increased lipid levels of paternal individuals may be confounded by lifestyles that promote hyperlipidemia. Additionally, this family appears to show an allele dosage effect where heterozygous carriers of a single putative pathogenic variant result in mild signs/symptoms, especially macrothrombocytopenia.

Hematological alterations have long been noticed in individuals with sitosterolemia, such as decreased platelet counts, increased mean platelet volume, and/or hemolytic anemia (Wang et al., 2004; Patel, 2014; Ajagbe et al., 2015). In addition to hypercholesterolemia, the proband had large platelets. Sitosterolemia can cause both phenotypes. Unexpectedly, all heterozygotes for Var2 in the maternal lineage exhibited large platelets without any changes in blood lipid profile. This is the first study to report a sign or symptom caused by heterozygosity for an *ABCG5* variant.

It has been long noticed that homozygous mutant mice with disruption of Abcg5 and Abcg8, Del (17Abcg5-Abcg8)1Hobb, have increased mean platelet volume (Yu et al., 2002). Nevertheless, this had not been previously reported in heterozygotes. We generated an ABCG5/8 double knockout rat, which illustrated that heterozygous *ABCG5* variants can lead to large platelets independent of hypercholesterolemia. Moreover, this trait was successfully corrected by Ezetimibe treatment in alone both the compound heterozygous proband and heterozygote II-4. Additionally, our preliminary experiment showed that Ezetimibe resolved both hypercholesterolemia and macrothrombocytopenia in two knockout rats. Taken together, Ezetimibe treatment can improve platelet production via unknown mechanisms.

Large platelets are usually accompanied by thrombocytopenia. Thus, it is also termed macrothrombocytopenia. Macrothrombocytopenia is usually due to acquired disorders; inherited large platelet disorders are rare. The mechanisms of large platelet formation and thrombocytopenia are poorly understood. Treatment for acquired versus inherited forms differs. Several different genes have been implicated as causes (Saito et al.). The most common clinical manifestation of inherited large platelets includes bleeding tendency (Mhawech and Saleem, 2000). Platelet count is usually decreased along with their increased size, which explains the abnormal blood coagulation. Overall, Var2 carriers other than the proband seem to be asymptomatic, similar to a few cases exhibiting large platelet syndrome. It is noteworthy that individual I-3 exhibited cerebral ischemic stroke at the age of 58, with normal blood pressure and lipid profile. Particularly, previous studies pointed out the large platelets may increase the stroke risk (O'Malley et al., 1995; Bath et al., 2004). Recently, it has also been reported that heterozygous carriers of ABCG5 variants had a 2-fold increase in the risk of coronary artery disease (Nomura et al., 2020). Therefore, determining the disease risk of Var2 carriers may require future evaluation in cohort studies, as a potential benefit for Ezetimibe treatment in these carriers.

Frequencies of pathogenic variants of *ABCG5* should be quite low in the general population; by July 2021, pathogenic or likely pathogenic variants have been reported for 66 cases in ClinVar, and the allele frequency is about 0.04% (126/282856) in gnomAD (2.1.1). The present study, however, found a high prevalence of *ABCG5* variants in a general population with large platelets. Screening for large platelets, therefore, may be helpful to identify heterozygotes for *ABCG5* pathogenic variants; conversely, sequencing *ABCG5* in individuals with large platelets may be useful in identifying patients amenable to Ezetimibe treatment, since a heterozygous family member with large platelets responded to this treatment.

Generally, physicians may only review the average volume/size of platelets, such as mean platelet volume (MPV), examined by the automated blood cell counting systems. But they may commonly ignore an abnormal “NaN” value of MVP in the system report, which stands for a biased platelet size distribution, particularly when patients have normal functions of blood coagulation as our observation in this pedigree. Here we showed the abnormal “NaN” value of MVP stood for the occurrence of large platelets over 30 fl, which can be the only symptom for some carriers of *ABCG5* variants. In addition, this volume is equivalent to spheres 4 microns in diameter to identify large platelets, which we adopted at the criteria in the blood film examination. Our study suggested the review of abnormal platelet size distribution may help to identify these carriers more easily. Meanwhile, they may have an increased risk of other complications such as stroke. Further larger studies could incorporate improved methods for detection of large platelets, identification of *ABCG5* variants in those with large platelets, the risk of clinical complications in such patients, and the effects of Ezetimibe treatment.

## Conclusion

In summary, WGS and plant sterol analysis of a family trio identified sitosterolemia in a proband with recalcitrant hypercholesterolemia and macrothrombocytopenia; she was found to have compound heterozygous variants in the *ABGC5* gene. Diagnosis confirmed in this manner guided appropriate therapeutic decisions, leading to clinical improvement. Interestingly, the therapeutic regimen not only reduced the proband’s blood cholesterol level to normal levels but also resolved macrothrombocytopenia. In the course of the evaluation, *ABCG5* heterozygosity was identified as a cause of autosomal dominant inherited macrothrombocytopenia; the role of *ABCG5* in macrothrombocytopenia and its therapy with Ezetimibe should be further investigated.

## Data Availability

The detailed methods described above and other measurements/assays for plasma cholesterols and platelets were available in the Supplementary Appendix of the full text of this article. WGS and Gene panel raw data are available from the corresponding author on reasonable request with consent from the patient family. VCF files for variants in ABCG5 genes and the Gene panel are publically available at https://github.com/humangenetest/ABCG5variants.

## Abbreviations

ABCG5, ATP-binding cassette transporters G5; ABCG8, ATP-binding cassette transporters G8; NPC1L1, Niemann–PickC1-Like1; LDL-C, low-density lipoprotein cholesterol; HDL-C, high-density lipoprotein cholesterol; WGS, whole genome sequencing; FH, familial hypercholesterolemia; GATK, Genome Analysis Tool Kit; ACMG, American College of Medical Genetics and Genomics; MPV, mean platelet volume; PLT, platelet; fL, femtoliters.
