# Supplementary material for: Remediation of ABCG5-Linked Macrothrombocytopenia With Ezetimibe Therapy
Source: Front Genet. 2021 Nov 22;12:769699. doi: 10.3389/fgene.2021.769699 (PMC8645579; doi:10.3389/fgene.2021.769699)
Supplement: Supplementary file 1 [file DataSheet1.doc]

**Supplementary Appendix**

**TABLE OF CONTENTS**

**I. Supplementary Figures**

1. Haplotype sequence of the proband in the region including Var1 and Var2
2. The size distribution of platelets (PLT) and the blood smear for the I-3, I-4, II-2 and II-3
3. Monitoring of the platelets for the II-4
4. Growth curves (A) and ABCG5/ABCG8 expression (B) in wild type (WT) and knowout (KO) SD rats
5. The size distribution of platelets (PLT) for 30 individuals with giant platelets.

**II. Supplementary Tables**

1. Primers of customized Gene Panel Test for 4 common FH causal genes
2. Primers and PCR protocols for amplification of ABCG5 exons and splice regions
3. Summary of all reported cases with ABCG5 deficiency
4. Phenotype survey of the proband
5. 9 variations identified in 30 individuals with abnormal platelet distribution

**IV. Supplementary References**

**I. Supplementary Figures**

**Figure S1. Haplotype sequence of the proband around the region including Var1 and Var2**

**
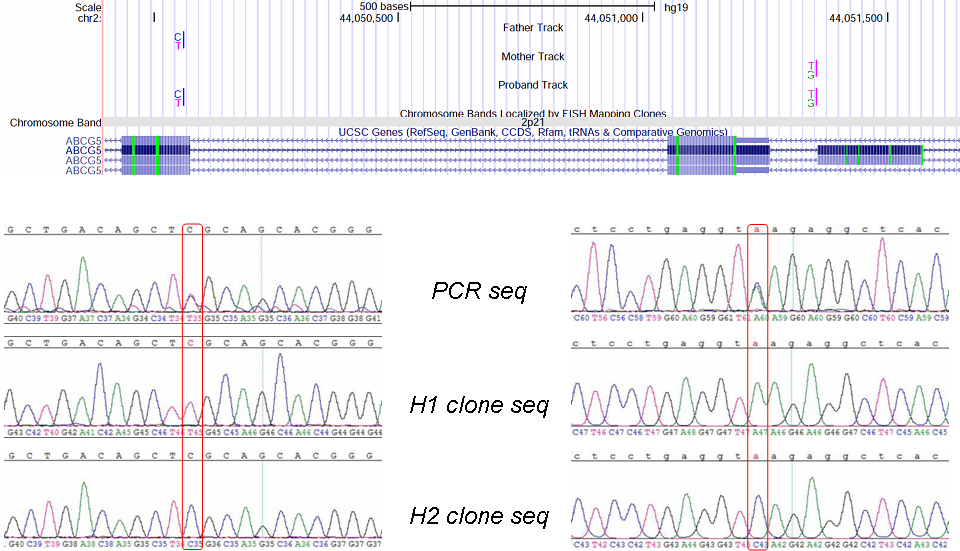
**

**Figure S2. The size distribution of platelets (PLT) and the blood smear for the I-3, I-4, II-2 and II-3.** The dash lines demonstrate the volume for the platelets, and the double dash lines show the 30 fL.

**Figure S3**. **Monitoring of the platelets for the II-4.** (A) Comparison of platelet size distribution before and after treatment. (B) Disappearance of giant platelets in blood smear for the II-4 after the treatments.

**Figure S4 Growth curves (A) and ABCG5/ABCG8 expression (B) in wild type (WT) and knowout (KO) SD rats.**

**
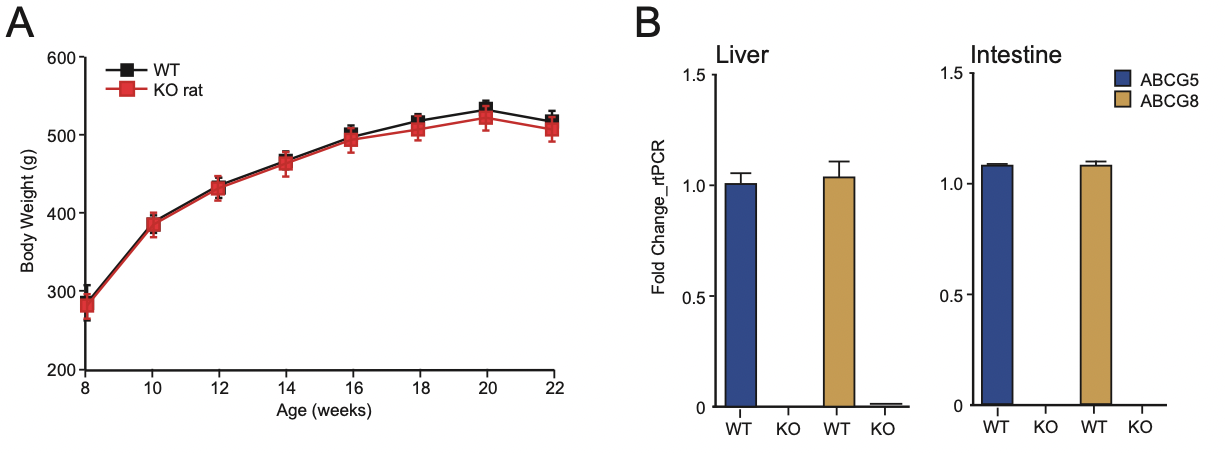
**

**Figure S5** The size distribution of platelets (PLT) for 30 individuals with giant platelets.

**
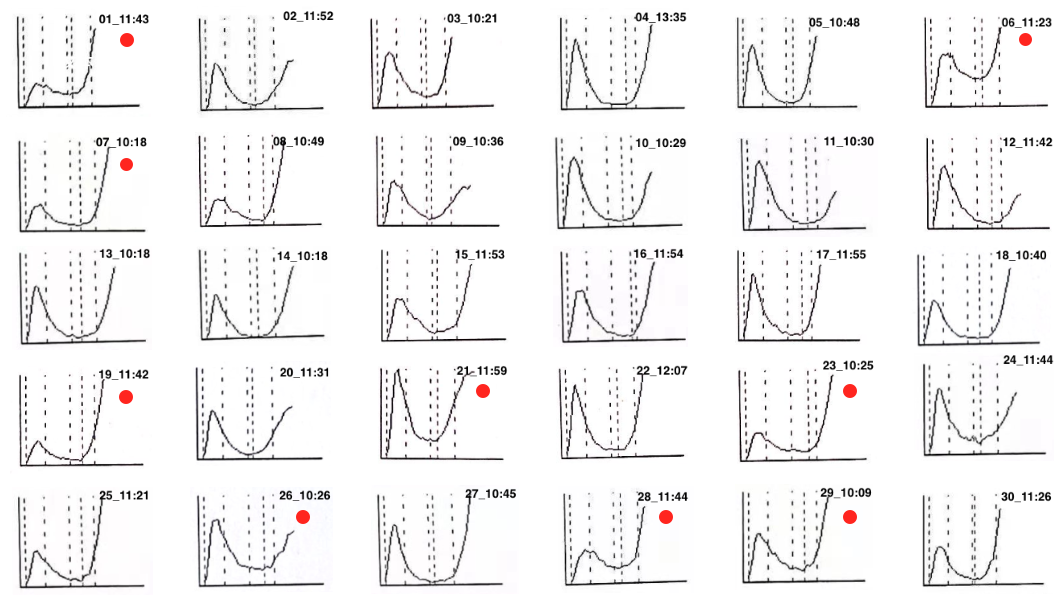
**

Red dots indicate the cases for which instrument could not export values for all parameters of the platelets

**II. Supplementary Tables**

**Supplementary Table 1.** Primers of customized Gene Panel Test for 4 common FH causal genes

| **Amplicon_ID** | **Ion_AmpliSeq_Fwd_Primer*** | **Ion_AmpliSeq_Rev_Primer*** | **Symbol** | **Chr** | **Amplicon_Start** | **Amplicon_**  **Stop** |
| --- | --- | --- | --- | --- | --- | --- |
| AMPL3677489788 | GGTGGTTACATCCAGCTTTAGGAA | CCTGAATGCTAACACTAAGAACCAGA | APOB | chr2 | 21228658 | 21228877 |
| AMPL4702519983 | CTTGGTTTTCTTCAGCAAGGCTTT | CCCTTTGGTGCTCTGATTAGAGATTAAG | APOB | chr2 | 21263842 | 21264053 |
| AMPL4702563407 | CAAGGGCAGACAGTGGCTAT | AGTCCTTCACAGGCAGATATTAACAAAA | APOB | chr2 | 21251109 | 21251326 |
| AMPL4702569339 | AGGGTATCCACCAAGGCTCT | GAGGCTGAATTGTTTTGGGAACATAA | APOB | chr2 | 21241887 | 21242066 |
| AMPL4702572764 | GCTGATCCCTCGCCATGTT | CTTTCTTCTCAGCCCCATCACTTT | APOB | chr2 | 21255269 | 21255465 |
| AMPL587079551 | GCAAATGATGAAGTTCTCAGCTTCTTAT | CATTATTCCTGGGCTGAAACTAAATGA | APOB | chr2 | 21228401 | 21228550 |
| AMPL587080662 | TGGTGAATGGAGACACTTCAACATT | GCTGTGCTTTGTGAGTTTATCAGTCA | APOB | chr2 | 21229947 | 21230169 |
| AMPL587110603 | TGCATCAATGACAGATGAAGATGAAGAA | CCAGTCCTTCATGTCCCTAGAAATC | APOB | chr2 | 21229612 | 21229827 |
| AMPL587118187 | CATAGAGAGAAATCTTTCAGTGGAGGAG | TCTGCTGGAAACAACGAGAACAT | APOB | chr2 | 21230282 | 21230433 |
| AMPL587123574 | AGTCAGTGCTTGAAAGGAAGGAA | GATAACCGTGCCTGAATCTCAGT | APOB | chr2 | 21228088 | 21228304 |
| AMPL587126935 | GGATGCAGTACTACTTCCACTTTTGTTA | CAACTGCAGACATATATGATACAATTTGATCAG | APOB | chr2 | 21232999 | 21233223 |
| AMPL587127039 | TGATTTTGGCATCATCTAATGCAATTTGT | CCCACAGCAAGCTAATGATTATCTGAA | APOB | chr2 | 21233244 | 21233386 |
| AMPL4702574822 | GCATAGCTCACCTTGCACATTGTA | CCACGAATGTCTACAGCAACTTG | APOB | chr2 | 21236021 | 21236172 |
| AMPL4702588225 | TGAGTAGATTTTCCAGCAACTATGTGG | CAGGGAAAACCTTCAACATGGAAAG | APOB | chr2 | 21257647 | 21257848 |
| AMPL4702597728 | GGAACTTGACCATTAACCCAGTACAAA | CTGATGCCATTTTCTTGTCTGATTTTCT | APOB | chr2 | 21249724 | 21249871 |
| AMPL4702603581 | CACATTGCCCTTCCTCGTCTT | GATTCCTTCTGCTTTTTCTTTCACGAT | APOB | chr2 | 21260068 | 21260231 |
| AMPL4702622694 | GAGGTCATGGAGACTGGCAAA | TCCCACATTGGGAACTGAAAGG | APOB | chr2 | 21247865 | 21248068 |
| AMPL4702622904 | GGCTGGTGTCCTAACAGTCTCT | AATATTGTTGTGAAGGTCAGAGGCA | APOB | chr2 | 21236288 | 21236489 |
| AMPL4702631687 | GTGCAGTAATTCAGGCCAGGAA | GCACCCTGAGAATTTGTGATGTC | APOB | chr2 | 21242664 | 21242851 |
| AMPL4702639864 | CAGTGTGTACTGACAGGACTGG | TCCTGCTCCCTTCTACCTTGAA | APOB | chr2 | 21258527 | 21258667 |
| AMPL4702643442 | CAGACTTGGCTGAAAGAATTACCC | GAGTTTGTGACAAATATGGGCATCAT | APOB | chr2 | 21245681 | 21245875 |
| AMPL4702734718 | TCTGTGATCCAGGAGTCTATTAGCA | CCAAAGTAATGCAGGTGTTGAGAC | APOB | chr2 | 21237996 | 21238211 |
| AMPL4702739643 | GCATCCTCCATACCTTGCAGTTG | CCCCTCTCTGCCCAGAATTG | APOB | chr2 | 21265220 | 21265425 |
| AMPL587139369 | CCTCTCAAAATATTCTTGCAAGGTCTCA | GACCTAACTCTACTAGACTCCCCAAT | APOB | chr2 | 21233491 | 21233694 |
| AMPL4233140585 | CTGTAACTCTTGAGAAGCTTCCTGAA | AAGATAACATTAAACAGCTGAAAGAGATGAAAT | APOB | chr2 | 21225169 | 21225362 |
| AMPL587144133 | AAAAGCACTCTAACATCAATAGCCTCAA | GCATCCTGGATTCAAAATGTGGATACTA | APOB | chr2 | 21232847 | 21233004 |
| AMPL587156123 | GCTAAGCTTGTGGTCAACTGCT | AGTACTGTGAGCTTAACCACGAAAAAT | APOB | chr2 | 21229294 | 21229506 |
| AMPL587182477 | TGTTCAGACTGTTTGTGTGGTCAA | GCTTCAGGGAACACAATGCAAA | APOB | chr2 | 21234465 | 21234674 |
| AMPL587201708 | CTTCATATCTTCCTGTTATCTGGTTGGT | GCTTCAGTTCATTTGGACTCCAAAA | APOB | chr2 | 21235125 | 21235341 |
| AMPL587214973 | CTGGGTCAAGTGATGGAAGAGA | GGGTGCAAGAGTCAAAGGATAAT | APOB | chr2 | 21250816 | 21251001 |
| AMPL587228279 | CCAATCCTTAGTGTCGCCTTGT | AATGGGAAGTATAAGAACTTTGCCACTT | APOB | chr2 | 21234797 | 21235017 |
| AMPL587239776 | ACCTCTGTGGTCTTGAGAGACT | CATAAGGGAGGTAGGGACGGT | APOB | chr2 | 21225410 | 21225634 |
| AMPL587243150 | ATTCTTGAGTAACTCGTACCAAGCC | GGGCCATTAGGCAAATTGATGAT | APOB | chr2 | 21225750 | 21225941 |
| AMPL587244175 | CTTTGGTGCAGGTCCAGTTCATA | AACCACAGTCATGAACCCCTAC | APOB | chr2 | 21224438 | 21224662 |
| AMPL587244614 | TGTGCAGAAATTGCTCAACTTGAC | GGCTGGACAGTGAAATATTATGAACTTGA | APOB | chr2 | 21224958 | 21225111 |
| AMPL587253207 | TCTAGAGTCTCATTCCCCTAGTACCT | GTCCAAAGTACAAAGCCATCACTG | APOB | chr2 | 21252431 | 21252588 |
| AMPL4702760901 | TGAATGTCCAGGGTGAGTCTGTAA | CAAAACATAGCTTCTTACCACACATCTC | APOB | chr2 | 21239353 | 21239574 |
| AMPL587253845 | CCGTGATGTATATCAGAAATGTGTGGTA | CTTTCTGCCACTGCTCAGGAA | APOB | chr2 | 21224685 | 21224889 |
| AMPL587271935 | CCAGGAAAAGTGCTTCTGAAATGATGTA | GGGTTACCAAGTCCTGACTCT | APOB | chr2 | 21252718 | 21252925 |
| AMPL587273518 | CTTGGGCACGTTGTCTTTCAG | TTTTCTCTCTGTTTAGTCCTCTCCAGAT | APOB | chr2 | 21226066 | 21226222 |
| AMPL637590428 | AAGTTGCTTACCGCCTGTCT | GGATATGGATGAAGATGACGACTTTTCTA | APOB | chr2 | 21227015 | 21227195 |
| AMPL4653086575 | GACAGACCAGGCTGACATTTTC | CCTCCCTCCTGAAAGGTGAAAC | APOB | chr2 | 21266388 | 21266529 |
| AMPL637979996 | TGTGGCGTAGAGACCCATCATAT | GCACAATTGGCCCAGAAAAATGG | APOB | chr2 | 21235455 | 21235634 |
| AMPL1012820289 | GGCAAAGAACTGTTCTACTTAGTAGCA | GAGTCTGCCAGTCAATAAATACATGGAT | APOB | chr2 | 21237715 | 21237936 |
| AMPL1012976280 | CCACTACTGGAGCTAGTGAGGTA | CAAGGCTGGAGTAAAACTGGAAGT | APOB | chr2 | 21246205 | 21246427 |
| AMPL1226567828 | TGACACTAGATTTTCTACAGTTTGGTTT | CTCAACCGTACAGTTCCTAGAATATGA | APOB | chr2 | 21227770 | 21227986 |
| AMPL1498067538 | GATTGATATCCAAATGGTCCCTGAGA | AGTCACATCTCTCTTGCCACAG | APOB | chr2 | 21256066 | 21256209 |
| AMPL1500364378 | GGAAGTGCCTGGTGGTTCTTA | CATGCTGAAAACTACCCTCACTG | APOB | chr2 | 21250587 | 21250754 |
| AMPL4701702467 | GCTTTTCCTTCCCATTCCCTGAA | AGTATGGAGCTTTTATTGAATTGAAACCT | APOB | chr2 | 21227307 | 21227433 |
| AMPL4701702474 | AAAAGTCATTCTTTGAGCCCTTCCT | CTCTCCAATGTATGGGAGGTTGTAATT | APOB | chr2 | 21246525 | 21246649 |
| AMPL3676958730 | GCTTTGCTTGTATGTTCTCCGTT | AGTGAGGCCAACACTTACTTGAATT | APOB | chr2 | 21228980 | 21229197 |
| AMPL3677041254 | CAGGTGTCTGGAAGGTAGCTTT | GGTTTATAGCACACTTGTCACCTACA | APOB | chr2 | 21231894 | 21232117 |
| AMPL3677066071 | GCTATCCAGGGTAAGCTGATTGTTTA | GAGTCAGTGAAGTTCTCCAGCA | APOB | chr2 | 21231085 | 21231264 |
| AMPL3677151632 | AGAACTTCTAATTTGGACTCTCCTTTGG | CTTCCCCACATCTCACACACAA | APOB | chr2 | 21231329 | 21231513 |
| AMPL3677154866 | CCCTGTTAACCTTAATGGAAAACGAACT | CTAAAGGCATGGCACTGTTTGG | APOB | chr2 | 21230542 | 21230729 |
| AMPL3677216852 | AAGCAGGGCACTGACTTTGT | CTGTAATGGCCCCGTTTACCAT | APOB | chr2 | 21233824 | 21234047 |
| AMPL3677248777 | AGGGTGATTCTCGCTAGAGGAAT | TCCACACCAGAATTTACCATCCTTAAC | APOB | chr2 | 21231617 | 21231804 |
| AMPL3677250584 | TTGAGGGAGCCAGATTCATAAACC | TGGCCACATAGCATGGACTTC | APOB | chr2 | 21230795 | 21230992 |
| AMPL3677272201 | AAGCCTCCTGTAACCAATTGATGATTAA | ATTACCACCAGTTTGTAGATGAAACCAA | APOB | chr2 | 21232242 | 21232448 |
| AMPL3677303433 | GACAGCATCATCAATAAATCCAACCAATT | TCAGAGCCAAAGTCCATGAGTTAATC | APOB | chr2 | 21232537 | 21232730 |
| AMPL4703594094 | GAGCCATAAGCTGTAGCAGATGA | CAAATGGAACTTCTGTTTAATCCTGCAT | APOB | chr2 | 21238274 | 21238498 |
| AMPL4703615584 | CTGGATATTTTGCTCAGAGATGGTTAGT | CAATTTCCAGCTTCCAGTAGACAAAGATA | APOB | chr2 | 21256266 | 21256469 |
| AMPL4703636088 | CCTTGCTGCTTTCTTCTTTTACCTTTTT | CATCTGGGAGTCTTCCTTATACCC | APOB | chr2 | 21237298 | 21237440 |
| AMPL4703648744 | AGAAATGATGCCCCTCTTGATGTTC | CTTCAGTGCCACCCAGCTTA | APOB | chr2 | 21260881 | 21261061 |
| AMPL4703666536 | TGTGTTGAGCCGATGGCTAAA | ACCTGAAATACAATGCTCTGGATCTC | APOB | chr2 | 21234130 | 21234341 |
| AMPL3677036261 | CCATTTCCATGACCCTTTTCCAGAA | GAGCTTAGTAATGGAGTGATTGTCAAGA | APOB | chr2 | 21230947 | 21231138 |
| AMPL3677110305 | GCTCACTGAGTAAAAGTGGCACTTTA | GCATCAGTGCAGCTCTTGAAC | APOB | chr2 | 21233643 | 21233864 |
| AMPL3677111451 | CATGCTCCGTTCTCAGGTACT | GGTATCGCAGCTTCCATCACTG | APOB | chr2 | 21231222 | 21231378 |
| AMPL3677168109 | AGCAAGAGTCCACCAATCAGAAA | AGCCTACAGGACACCAAAATAACC | APOB | chr2 | 21232069 | 21232293 |
| AMPL3677196226 | CAGTAAACTCTGCCTTCCCTTCT | GCAAACACCTAAGAGTAAACCAAAACTT | APOB | chr2 | 21230685 | 21230846 |
| AMPL3677218487 | AGCTTGCCAAAAGTAGGTACTTCAA | AGGGATCTGAAGGTGGAGGAC | APOB | chr2 | 21231467 | 21231660 |
| AMPL3677282654 | AATTGTAAAGGAAGGAATGTGGAAGGT | GAAGTCAGTCTTCAGGCTCTTCAG | APOB | chr2 | 21231751 | 21231939 |
| AMPL3677289611 | GAGTCACCTCACGGATTTTGTCA | GCTAAGCAATGTCCTACAACAAGTTAAGA | APOB | chr2 | 21232398 | 21232612 |
| AMPL3677311738 | GGATTTGTTGGTCTACTTCATACCTCTC | ACCTAGCTGGAAAGTTAAAACAACACA | APOB | chr2 | 21232677 | 21232901 |
| AMPL3677499003 | CCGGACTTCATTTTTCCATCTGATCT | AGCTAGAGGGCCTCTTTTTCAC | APOB | chr2 | 21228826 | 21229025 |
| AMPL3766439257 | GGGTTCAGTTTTAATACAGAGATGCAC | AGCAGCTGCGAGAGATCTTC | APOB | chr2 | 21255143 | 21255307 |
| AMPL587069627 | CATTTATTCCTACATGGGCCTCCATA | CATCCACAAACAATGAAGGGAATTTGAA | APOB | chr2 | 21230385 | 21230597 |
| AMPL587074088 | GGAGGCAGGATATTTCTTACCATTTAGT | CTCTGTACCTGCTGGAATTGTCA | APOB | chr2 | 21227932 | 21228133 |
| AMPL587080616 | TGTTGCCACTGACACTTCCAT | CAGTCAGATATTGTTGCTCATCTCCT | APOB | chr2 | 21229459 | 21229665 |
| AMPL587096945 | TGCCTGTCAAAGGATTTGATGCT | ATGCGTCTACCTTACACAATAATCACAA | APOB | chr2 | 21230120 | 21230337 |
| AMPL587113071 | CGTAGGCATGACAAGAACTGAATTTAGA | CAGTCTATGACAAGAGCTTATGGGAT | APOB | chr2 | 21228496 | 21228707 |
| AMPL587129104 | CCTTGAAATCTGGAAGAGAAAGCTTGA | CCTGGATACACTGTTCCAGTTGT | APOB | chr2 | 21229776 | 21229995 |
| AMPL587132430 | TGAAACTTGTCTCTCCCAATTGAATGA | TGTTCACTCCATTAACCTCCCATTTTT | APOB | chr2 | 21233332 | 21233545 |
| AMPL587161788 | GCGTGAACTGGGACACAGTTA | GTGCAAACTTGACTTCAGAGAAATACAA | APOB | chr2 | 21228261 | 21228460 |
| AMPL4702558855 | AAGGAAGATTATCTGCTAGAAAGCCA | TCTGTGGTTGACCTGCTTTCC | APOB | chr2 | 21235921 | 21236065 |
| AMPL4702567381 | CTGTATCACTTGTTAGTCAGCAGACAT | CTTCTTTGGTGAAGGTAAGAGTTTCTGT | APOB | chr2 | 21257577 | 21257701 |
| AMPL4702591921 | CCACACTGAACCAAGGCTTGT | TGATTAAGGGCTCTCCTGGCA | APOB | chr2 | 21255421 | 21255549 |
| AMPL4702597715 | TGTTCCCATGGTAGAATTTGGACAA | GCTAGCAGACAATGCCAGTGT | APOB | chr2 | 21251274 | 21251495 |
| AMPL4702600052 | AGGAGGCGGACCAGTTGTA | GCAAATCCTCCAGAGATCTAAAGATGTT | APOB | chr2 | 21236131 | 21236337 |
| AMPL4702600811 | TTTTCCTTCCAAGCCAATCTGAGAA | TGGTATTCAAATACTTGTTTAGATAAATGGACA | APOB | chr2 | 21249819 | 21249945 |
| AMPL587184183 | GCTTAGAGAAGGTCATATCCATCTTGTT | AGGTTTAACTCCTCCTACCTCCAAG | APOB | chr2 | 21234960 | 21235179 |
| AMPL587200225 | GCCGTAGTTTCCCATTGTTGGT | TGATGGGCTCATATGCTGAAATGAAAT | APOB | chr2 | 21234294 | 21234515 |
| AMPL587204596 | GGCTTTCCCATCCAGACTGAAT | GCACTGACAAAATTAATAGTGGTGCTC | APOB | chr2 | 21234631 | 21234845 |
| AMPL587207454 | GTCAGCTGAAGCAAATCCAAAGG | CTCGGAACTATCAACTCTACAAATCTGTT | APOB | chr2 | 21250709 | 21250866 |
| AMPL587218513 | TTGACTTCTTTGACAAACAAATGCTGTT | TGACCACAAGAATACGTTCACACTATC | APOB | chr2 | 21235286 | 21235504 |
| AMPL587237827 | CGCAATGGCCTGGCTTTTAATTAT | CCTCTAACTTTACTTCCCAACTCTCAA | APOB | chr2 | 21224845 | 21225008 |
| AMPL587238021 | TGAAGATTGTGTTGATCTCATCTTGGAT | AGCCCAAGAGGTATTTAAAGCCATT | APOB | chr2 | 21225282 | 21225457 |
| AMPL587244227 | GACTTTCGAATATACCTGGGACAGT | GGGACTCAAGGATAACGTGTTTGAT | APOB | chr2 | 21225589 | 21225799 |
| AMPL587251003 | GTTCTCCTGGAGCAAGCTTCAT | CAAAAGATTGATTGACCTGTCCATTCA | APOB | chr2 | 21224619 | 21224743 |
| AMPL587258669 | CTTTCTGGAACCTCACGTCGAT | AGCTTCTGGCTTGCTAACCTC | APOB | chr2 | 21225897 | 21226108 |
| AMPL587261233 | TGAGGGCTGTAGTAGAAGTTCCATT | CTTTCGTGATAGCTCATCTGTTTTTCTG | APOB | chr2 | 21227142 | 21227359 |
| AMPL637430943 | AGAAGGCTAGTGACAGGGTCT | TGGTCAGTTTGCAAGCAAGTCT | APOB | chr2 | 21242483 | 21242707 |
| AMPL4702608977 | GTACAAGCATCTTTTCGGGCTT | TCTTGGGAGAGGAGCTTGGT | APOB | chr2 | 21247694 | 21247905 |
| AMPL4702615695 | TCCTCAGGACCTGAATGATCTCAA | CAGTCAAGCTGCTCAGTGGA | APOB | chr2 | 21245592 | 21245724 |
| AMPL4702625136 | GCCTGGAACAGAGCACTTGAG | TTGTCAACTCTGATCAGCAGCA | APOB | chr2 | 21258375 | 21258571 |
| AMPL4702659253 | CTCCTAGCGAAGTCCGGAATG | AAATCTTTGCCCGGAGGTGA | APOB | chr2 | 21245829 | 21246009 |
| AMPL4702694445 | ACTGAAGTCCTTCATATTTGCCATCTTC | GGTTAGGATAGAATTCTCCCAGTTTTCA | APOB | chr2 | 21227431 | 21227638 |
| AMPL4702714942 | AACATGGCTTGGTCAGGTATGAA | TGAATCTACTGAGGGCAAAACGTC | APOB | chr2 | 21239217 | 21239400 |
| AMPL637944367 | GACCCTCAACTCAGTTTTGAATATGGT | CCATAAAACAAACACATGAACTGACAT | APOB | chr2 | 21226159 | 21226368 |
| AMPL4233139900 | AAATTTACACGGATATGATAGTGCTCATCAA | ACAAATTGCATTAGATGATGCCAAAATCA | APOB | chr2 | 21233075 | 21233272 |
| AMPL1012216917 | GGCTGACTTACAAACAGAGGCA | GATTCAAGAAGTGCCACCAGGAT | APOB | chr2 | 21265056 | 21265265 |
| AMPL1012880407 | CTTCCTGACAGGGTTGGTGTT | CCACATCCCAGAAAACCTCTTCTT | APOB | chr2 | 21237128 | 21237349 |
| AMPL1013060206 | AGTATATTTTGAGCTGACACACCATGTT | GCTTCTGAAACAAACACTTGTGCAAAT | APOB | chr2 | 21250951 | 21251109 |
| AMPL4567386395 | TCCATGTATTTATTGACTGGCAGACTC | CCGATTATCCTAAGAGCTTGCATATGTA | APOB | chr2 | 21237910 | 21238048 |
| AMPL1498159057 | CATTAGATACCTGGACACCTCAATCAG | TTTGAAGACTCTCCAGGAACTGAAAAA | APOB | chr2 | 21256161 | 21256320 |
| AMPL4567917755 | TGTCTCTTAAGCTGCTGCAGTT | AAAAACAATCCATGATCTACATTTGTTTATTGA | APOB | chr2 | 21232925 | 21233071 |
| AMPL4652891859 | GGTGCCCACTAGCTCAAAAGTT | CTGCTCCACTCACTTTACCGT | APOB | chr2 | 21259887 | 21260110 |
| AMPL1637061913 | CAGGTTCTTGATCAGACTGACTATCTTT | AACGAGCTTCAGGAAGCTTCT | APOB | chr2 | 21225052 | 21225201 |
| AMPL4652934767 | CTGGTGCAAACACACAAGTTCA | GGTGTATGGCTTCAACCCTGAG | APOB | chr2 | 21263737 | 21263890 |
| AMPL4652943930 | TTTTGAGGACTTCCATGCTTAGAAA | GATTTTTCCCAGACAGTGTCAACAAAG | APOB | chr2 | 21249574 | 21249778 |
| AMPL4653069015 | GCCATCTCAGCCCTGTAGAGT | CCTGTCTTACAGAAGAGGAAATGCTG | APOB | chr2 | 21266285 | 21266435 |
| AMPL4703586227 | TGTGAATGGTACTAGTTCAGCCTGTA | CCAAACTGCTTCTCCAAATGGAC | APOB | chr2 | 21238155 | 21238319 |
| AMPL4703597808 | GGATGGCAGCTTTCTGGATCAT | AGCACTTTTCCTGGACACCTTTTA | APOB | chr2 | 21252543 | 21252731 |
| AMPL4703623413 | TCATGGAGCTGACTCAGTGATCT | CGGAGAAAGATGAACCTACTTACATCCT | APOB | chr2 | 21260726 | 21260933 |
| AMPL4701683945 | GCAAACAGAATCTTACGTTGGCT | GGTCTACAGATTGGAGAGGTCATC | APOB | chr2 | 21246381 | 21246573 |
| AMPL3676976464 | CATGTCTTCTCCTCATGAATTCTGAA | CCTATGAGCTCCAGAGAGAGGAC | APOB | chr2 | 21241791 | 21241929 |
| AMPL3677020586 | ACTGAAGACCGTGTGCTCTTG | AATGCTGTACTCTACCGCTAAAGG | APOB | chr2 | 21229151 | 21229339 |
| AMPL4703642609 | CTATTGAGGTGGTCTTGCAAAGTCT | GGTATTTTTGCCTAATGTTCATTGCTCT | APOB | chr2 | 21237392 | 21237543 |
| AMPL4703654789 | CCATTTGTATGTGCATCGATGGT | GCTAAGGTTCAGGGTGTGGAG | APOB | chr2 | 21234002 | 21234171 |
| AMPL413502387 | CCCTGACTCCGCTTCTTCT | CGCAGAAACAAGGCGTGTG | LDLR | chr19 | 11231020 | 11231223 |
| AMPL413542372 | ATTCTTTAGTTGGCAGGAAATAGACACA | GGACTCACAGCACGTCTCC | LDLR | chr19 | 11210808 | 11211029 |
| AMPL413576067 | CGGCGAAGGGATGGGTA | GCACTCGTAGCCGATCTTAAGG | LDLR | chr19 | 11221268 | 11221401 |
| AMPL413629258 | CGAAGATGGCTCGGATGAGT | CATACCGCAGTTTTCCTCGTC | LDLR | chr19 | 11216101 | 11216281 |
| AMPL413640942 | TGAGAAGTAGGTGGCCTCCA | TGCTTTGGTCTTCTCTGTCTTTGAATAA | LDLR | chr19 | 11241881 | 11242080 |
| AMPL413738884 | GCCTGAATGGTGTGGACATCT | GAAACCTTCAGGGAGCAGCTT | LDLR | chr19 | 11226820 | 11226996 |
| AMPL3989014358 | TCAGTGGGTCTTTCCTTTGAGTG | CGTTGTCGCAGTCCACTTG | LDLR | chr19 | 11213291 | 11213441 |
| AMPL3989324171 | AAAGGCCCTGCTTCTTTTTCTCT | CCAACTTCATCGCTCATGTCCTT | LDLR | chr19 | 11217171 | 11217352 |
| AMPL3989544606 | GGTGGCCAGCAATAGAATCTACT | TGATGACGGTGTCATAGGAAGAGA | LDLR | chr19 | 11224072 | 11224260 |
| AMPL3989689733 | TTCCCGTTGGGAGGTCTTTTC | TGTACTCGCTCCGGTCCA | LDLR | chr19 | 11223802 | 11224025 |
| AMPL3989797055 | CGCAAGGCGATCTCTAAACAAA | GTCAAAGTTGATGCTGTTGATGTTCT | LDLR | chr19 | 11240052 | 11240271 |
| AMPL3989824420 | CGTCATTAGGCGCACACCTAT | GTGTCCTTACGGCTGTGGAG | LDLR | chr19 | 11233776 | 11233924 |
| AMPL4055019071 | CACAGACTTGGGAAGTTCTCCAA | CAGAGCCCTCACGCTACTG | LDLR | chr19 | 11238547 | 11238742 |
| AMPL4137497464 | AGCGTCCCCGGCTATAGAA | GGTCTGAGTCACAGACGAACTG | LDLR | chr19 | 11215817 | 11215976 |
| AMPL4137530416 | GGAATCAGAGCTTCACGGGTTA | AGTGTCCCGACCCGGAT | LDLR | chr19 | 11199980 | 11200204 |
| AMPL4703442186 | CTTCGAAGGTGTGGGTTTTGG | CCTTCCTCACACTGGCACTTG | LDLR | chr19 | 11222096 | 11222274 |
| AMPL4703449287 | GAGAGAGGGTGGCCTGTG | ACAGTAGGTTTTCAGCCAACAAGTT | LDLR | chr19 | 11230699 | 11230864 |
| AMPL4702062301 | TCAGCACGTGACCTCTCCTTA | CTCGTACGTAAGCCACACCT | LDLR | chr19 | 11227497 | 11227692 |
| AMPL4703470320 | GGACTGGATCCACAGCAACAT | TCCTGAAGCTCCTTCCTGCT | LDLR | chr19 | 11224295 | 11224500 |
| AMPL4702067023 | AATCCATTTGCATGCGTTCTTATGT | CAGTCTCTAGCCATGTTGCAGA | LDLR | chr19 | 11217957 | 11218155 |
| AMPL413600486 | CACGATGGGAAGTGCATCTCT | GGAACACGTAAAGACCCCTACA | LDLR | chr19 | 11215931 | 11216153 |
| AMPL413602187 | CTAGGACACAGCAGGTCGT | TCCCAGGGATGGAGTGATTATTTGTA | LDLR | chr19 | 11200168 | 11200392 |
| AMPL413639801 | GAATGCATCACCCTGGACAAAG | CCTACAGCACTCATGTCTCAGT | LDLR | chr19 | 11218112 | 11218263 |
| AMPL413643442 | TGAACCTGGAGGGTGGCTA | GTGCAAAGTTCAGAGGATGAAACTC | LDLR | chr19 | 11222235 | 11222401 |
| AMPL413649256 | CCCGACTGCAAGGACAAATCT | CGGCACCTAAATCACTGCATGT | LDLR | chr19 | 11216240 | 11216365 |
| AMPL413662091 | GGGTTCCCAGCAGGACTATTT | TGTTTTCAGTCACCAGCGAGT | LDLR | chr19 | 11226646 | 11226861 |
| AMPL414667929 | TCAGTTCTGGAGGTGCGATG | ACCCCGTAGAGACAAAGTCAGA | LDLR | chr19 | 11213401 | 11213536 |
| AMPL4702074984 | GCTGGCAGAGGAAATGAGAAGAAG | TGTCCAGGAGAAAAAGTGAACAGG | LDLR | chr19 | 11238698 | 11238861 |
| AMPL4702081921 | CAGTGTGACCGGGAATATGACTG | CCCTCTGGCTTCACAAATCATTT | LDLR | chr19 | 11217306 | 11217441 |
| AMPL4702091595 | CCTTCTCCTTGGCCGTCTTTG | CCTCCTAGTCACAACCAGTTTTCTG | LDLR | chr19 | 11227652 | 11227777 |
| AMPL4702110420 | CACCGTCAGGCTAAAGGTCAG | CATCTCGTGACCAAAATGTTCGT | LDLR | chr19 | 11233884 | 11234102 |
| AMPL3988950525 | CCAGGATGGCTCTGATGAGT | GCCGCCATCATCAAAAAGGG | LDLR | chr19 | 11210987 | 11211210 |
| AMPL3989538556 | GCACGAGGTCAGGAAGATGAC | ATCATTCTCTGGGACAGGTCAGA | LDLR | chr19 | 11223985 | 11224119 |
| AMPL3989604425 | CATGAGGAGCTGCCTCACA | GTCAAGCCCGGTGCTGAT | LDLR | chr19 | 11231179 | 11231360 |
| AMPL4054977229 | TGTTCCCACGTCTGCAATGA | GTGGAATCTCATGAAACCCTCCTA | LDLR | chr19 | 11221360 | 11221580 |
| AMPL4055022853 | CTCTCCAGGTGCTTTTCTGCTA | AGGAGATCTAGACACACAAGTGGA | LDLR | chr19 | 11227406 | 11227541 |
| AMPL4137467001 | CTATGGAAGAACTGGCGGCTTA | GTCCCTTGAGGATCATATGCCT | LDLR | chr19 | 11240224 | 11240421 |
| AMPL4703455705 | AATGATCTGCAGGTGAGCGT | AGGACAGAGTCGGTCCAGTAG | LDLR | chr19 | 11224114 | 11224336 |
| AMPL4703467241 | GCCTCACAGGTTCCGATGTC | ACCAGAAGATTCCAGAAATTTCCAGAAC | LDLR | chr19 | 11230820 | 11230997 |
| AMPL4401981955 | CTGGGATGGAGCTGCAGAA | CAGCCTGTGCCTGAAAACAG | LDLRAP1 | chr1 | 25889004 | 25889144 |
| AMPL871354742 | GCTCTGCCTTCCAGGCTTG | CGCAGCAGGTGGCTTTG | LDLRAP1 | chr1 | 25893325 | 25893546 |
| AMPL871359545 | GTGGAGTGGGAATAGCAGGTT | CAAACACCTTGTCGTGCATCT | LDLRAP1 | chr1 | 25883531 | 25883687 |
| AMPL1417508083 | AGCCATTAGTCAGGTTCTCACTCT | ACTGTGAGGGATCCGGTCA | LDLRAP1 | chr1 | 25889962 | 25890152 |
| AMPL1417508102 | GGTTGTGTGGCCAGCAGAT | TGGGAGCCAAGCCTGTTG | LDLRAP1 | chr1 | 25890289 | 25890490 |
| AMPL1500485340 | TCCTAGGAAAGAAGGCTGGTG | ACTAGCGTCATGCCCAGGTA | LDLRAP1 | chr1 | 25880328 | 25880497 |
| AMPL3676940843 | TGGATGAAGCGTTTTCGAGGT | TCTGAGTGGCTTGTGTTCTTAATCAG | LDLRAP1 | chr1 | 25891680 | 25891806 |
| AMPL4702066737 | GGGCTTAGGAACAGTGAAGTGT | GAGGTTGTCTGTCAGGATAATTCCC | LDLRAP1 | chr1 | 25881266 | 25881428 |
| AMPL4702080257 | CTGAAACTGCCCCTTGAGGT | GCTGGCTTTGTCCCTCTTCT | LDLRAP1 | chr1 | 25889452 | 25889586 |
| AMPL4702068190 | GGGATGCTGTTCAGCCTCAAG | CAACTGGAGTAACAAGCTGGGA | LDLRAP1 | chr1 | 25880457 | 25880647 |
| AMPL871344379 | GCTGATCTCCCACTGACAACC | GAGGTCAGGCTGAGGATCAC | LDLRAP1 | chr1 | 25890113 | 25890329 |
| AMPL871380385 | GATCTCCTATTGCACAGCAGACA | GTAATGGTCACCTTTGGGTCCT | LDLRAP1 | chr1 | 25883644 | 25883865 |
| AMPL4401988444 | GGAGGTGCTTTGATCTGAGGTTA | CTGAGGGTTTGTCCGAGACTG | LDLRAP1 | chr1 | 25893180 | 25893366 |
| AMPL4401996594 | CCTCCCCATCCCCACTTC | CCACAATGTAGATGCAAAGTCTCAC | LDLRAP1 | chr1 | 25889107 | 25889232 |
| AMPL4402007081 | GCCTTGGTCCTGCAGAGAAAG | GAAAAACCAAGAGGCTCTCCCA | LDLRAP1 | chr1 | 25889546 | 25889748 |
| AMPL4402050903 | CAGAAGGTGACTCTGAAGGTGTC | CCCAAGTGGCAGAGTGGAT | LDLRAP1 | chr1 | 25881375 | 25881506 |
| AMPL1417508234 | GCCCTGACACGTGATGGAC | AGCATAAGCCAGAAGTCACTGAATAAAT | LDLRAP1 | chr1 | 25893511 | 25893720 |
| AMPL3676917086 | GCTTGTGTCCTGAGTCCCTGTA | CCAGCACAGGAAGCTAGCATT | LDLRAP1 | chr1 | 25891557 | 25891721 |
| AMPL413508536 | TTGCTGGGTTTCTTCCATGTCA | CTCATCTTCACCAGGAAGCCA | PCSK9 | chr1 | 55509468 | 55509688 |
| AMPL3712526399 | GCCAAGGTGCGGGTGTA | TCCCCAAGATCGTGCCAAG | PCSK9 | chr1 | 55505712 | 55505864 |
| AMPL413519077 | GAACTTCAGCTCCTGCACAGT | CCGTCCTCGTCCTCCTG | PCSK9 | chr1 | 55505397 | 55505617 |
| AMPL3712549622 | GAGCGGATTACCCCTCCAC | GGAGGTGCTGAGTCCCAAA | PCSK9 | chr1 | 55512271 | 55512440 |
| AMPL3713799125 | CTTAGAGGTGTGAGAGGAGGCT | AGACAGCATCATGGCTGCAAT | PCSK9 | chr1 | 55523514 | 55523731 |
| AMPL414595636 | GCCTACGCCGTAGACAACAC | GACAAGTCGGAACCATTTTAAAGCTC | PCSK9 | chr1 | 55529117 | 55529331 |
| AMPL1012467495 | CCAGATGAGGAGCTGCTGA | GCTGGAGTCTGGAGGATGGA | PCSK9 | chr1 | 55524252 | 55524446 |
| AMPL1013168806 | GCCTGATCAAGGAGCGAGAAAA | CCTCGATGTAGTCGACATGGG | PCSK9 | chr1 | 55512010 | 55512229 |
| AMPL1494547002 | GCTCAACTGCCAAGGGAAGG | ACAGCATTCTTGGTTAGGAGACATTAG | PCSK9 | chr1 | 55518421 | 55518570 |
| AMPL4703355908 | GTCTACGCCATTGCCAGGT | GGATCACACTTGTGAGGACCAA | PCSK9 | chr1 | 55525213 | 55525428 |
| AMPL4701788825 | AGGCCTGAGTCTGCCTCT | GCTGGAGGCACCAATGATGT | PCSK9 | chr1 | 55522933 | 55523126 |
| AMPL4701791645 | GTTGAGGCAGAGACTGATCCAC | GGGAGAGACTGTCAAGGTCACA | PCSK9 | chr1 | 55523758 | 55523955 |
| AMPL4701827905 | CAGAGTGACCACCGGGAAATC | CCCAGGAGTACAGCTGCAA | PCSK9 | chr1 | 55517995 | 55518185 |
| AMPL4701829390 | CTGTGCTGAGGCCACGA | GTGGCACAAACTGACACAGAAAAG | PCSK9 | chr1 | 55527096 | 55527308 |
| AMPL3677194655 | CAGGCCTGGAGTTTATTCGGAAA | ACCTACCTCGGGAGCTGA | PCSK9 | chr1 | 55521663 | 55521868 |
| AMPL413521968 | CCTCATGGGCACCGTCA | CCCTACACCCGCACCTT | PCSK9 | chr1 | 55505507 | 55505731 |
| AMPL3677193311 | GGGAGCAGGTCTCCCCAA | GTTGAGGACGCGGCTGTA | PCSK9 | chr1 | 55521620 | 55521760 |
| AMPL414611490 | GACGATGCCTGCCTCTACTC | CGACTCCTTCCAAAGCCAGAA | PCSK9 | chr1 | 55521824 | 55522014 |
| AMPL414643158 | ATGTCTTCCATGGCCTTCTTCC | ACTAAGCACAGTCCCCAGTGTATAT | PCSK9 | chr1 | 55509646 | 55509783 |
| AMPL3712540308 | GCTTCTGCAGGCCTTGAAGTT | GGTATTCATCCGCCCGGTA | PCSK9 | chr1 | 55512186 | 55512310 |
| AMPL422385088 | CATCCCAGGATGGGTGTCTG | AGGCACCCAGAGTGAGTGA | PCSK9 | chr1 | 55529264 | 55529454 |
| AMPL3713058928 | GTTGACTTTATGCTCATTCCCTCCT | TCGAAGTCGGTGACCATGAC | PCSK9 | chr1 | 55517901 | 55518044 |
| AMPL423279419 | CCTCCTCTCTCCTACCATGAACTA | CCACTCCTGGAGAAACTGGAG | PCSK9 | chr1 | 55524085 | 55524295 |
| AMPL3713751478 | CGCTGTGTGGACCTCTTTG | CGGCACAGACCCTGACTG | PCSK9 | chr1 | 55523076 | 55523257 |
| AMPL4137386932 | TTGAGTTGATCCTGTCTAGTCCCT | GTGTGGACGCTGCAGTTG | PCSK9 | chr1 | 55525077 | 55525268 |
| AMPL4137395318 | CATCACCATCTTTCACCATTCACC | TCATTGATGACATCTTTGGCAGAGAA | PCSK9 | chr1 | 55523675 | 55523805 |
| AMPL1012192084 | CCTGAATGGCACATTTGAAAGTGTTATA | CCCATGCAAGGAGGAACATGA | PCSK9 | chr1 | 55509290 | 55509510 |
| AMPL1012506113 | CACTTTGGCCTCACAGAAGGAT | CCGGCTCCTGACTACACAC | PCSK9 | chr1 | 55528941 | 55529155 |
| AMPL4701756358 | CATCCAGCCACCTGCTGATT | CTATGAGGGTGCCGCTAACC | PCSK9 | chr1 | 55518248 | 55518464 |
| AMPL4701822259 | GGTTTCCTAGCTCTTGCCTCAGA | GCACTGGTTGGGCTGAC | PCSK9 | chr1 | 55526937 | 55527130 |

**Supplementary Table 2.** Primers and PCR protocols for amplification of ABCG5 exons and splice regions

| **Primers for amplicons ABCG5** | | | | | **PCR Protocols** | | | | | |
| --- | --- | --- | --- | --- | --- | --- | --- | --- | --- | --- |
| **Primer ID** | **Sequences (5'to3')** | **Amplicon (bp)** | **Exon (bp)** | **Flanking region (bp)** | 94℃ | 94℃ | Tm | 72℃ | cycles | 72℃ |
| **ABCG5-E1 L:** | CTCTCCACCCGATCCACTAA | 580 | 283 | 297 | 5 min | 30s | 56℃ | 45s | 30 | 5 min |
| **ABCG5-E1 R:** | TCACTCTGTTTCCTGGAGCA |
| **ABCG5-E2 L:** | AACACGTTAGGAGCCTGTCC | 395 | 122 | 273 | 5 min | 30s | 56℃ | 45s | 30 | 5 min |
| **ABCG5-E2 R:** | TTGGTAGCAGTTCCATTCACA |
| **ABCG5-E3E4 L:** | CACAGAGGGTCTCGGGAAG | 499 | 315 | 184 | 5 min | 30s | 59℃ | 45s | 30 | 5 min |
| **ABCG5-E3E4 R:** | GAGTGACGAGCAAAGGGAAG |
| **ABCG5-E5 L:** | TGTGCTGCCTCTTTCATGTC | 299 | 133 | 166 | 5 min | 30s | 59℃ | 30s | 30 | 5 min |
| **ABCG5-E5 R:** | AAAGGGCCCAAAGTATCTGC |
| **ABCG5-E6 L:** | CTCAGGCTGAAAGCCAGTTT | 382 | 140 | 242 | 5 min | 30s | 59℃ | 30s | 30 | 5 min |
| **ABCG5-E6 R:** | CCTGGCCACTGGTACAAATC |
| **ABCG5-E7 L:** | TGCATCAGTGTTCCCAGAGA | 282 | 130 | 152 | 5 min | 30s | 59℃ | 30s | 30 | 5 min |
| **ABCG5-E7 R:** | CATCCAGGCAGAAGTCTGAG |
| **ABCG5-E8E9 L:** | AGAGAGGGAACCTGGAGAGG | 788 | 520 | 268 | 5 min | 30s | 59℃ | 45s | 30 | 5 min |
| **ABCG5-E8E9 R:** | AGGTGGGCATTAACCACAAA |
| **ABCG5-E10 L:** | GATGGGCAAAGTGTAGATCCTC | 400 | 139 | 261 | 5 min | 30s | 59℃ | 30s | 30 | 5 min |
| **ABCG5-E10 R:** | CCTCCTTCTATGACTAGGGAACC |
| **ABCG5-E11 L:** | CCCACTCACCACAAAGGATT | 390 | 186 | 204 | 5 min | 30s | 59℃ | 30s | 30 | 5 min |
| **ABCG5-E11 R:** | CCATAACCACTATCAGTTCTCTGG |
| **ABCG5-E12 L:** | TTTCTAGGCAACGGTATTTCTTT | 526 | 113 | 413 | 5 min | 30s | 56℃ | 45s | 30 | 5 min |
| **ABCG5-E12 R:** | CCAAGTGTCCCTTACCTGTTG |
| **ABCG5-E13 L:** | AAGCGCTTGGTAAATACTTGTT | 1000 | 838 | 162 | 5 min | 30s | 56℃ | 60s | 30 | 5 min |
| **ABCG5-E13 R:** | TGCCTATAAGTAAACTCAGCTGAAAG |

**Supplementary Table 3. Summary of all reported cases with *ABCG5* deficiency**

| **Proband** | **Homozygous/Compound heterozygous** | **Mutation Position** | **Nucleotide alteration** | **PMID** | **TC (mg/dl）** | **β-sitosterol (mg/dl）** | **stomatocyte** | **Megaloplastocyte** |
| --- | --- | --- | --- | --- | --- | --- | --- | --- |
| 1 | Homozygous | exon 6 | R243X | 11452359 | NaN | > 8 | YES | YES |
| 2 | Homozygous | exon 9 | R419H | 11452359 | NaN | > 8 | NaN | NaN |
| 3 | Homozygous | exon 9 | R389H | 11452359 | 267 | 18.98 | YES | YES |
| 4 | Homozygous | exon 3 | del exon 3 | 11452359 | NaN | > 8 | NaN | NaN |
| 5 | Homozygous | exon 9 | R389H | 11452359 | NaN | > 8 | NaN | NaN |
| 6 | Compound heterozygous | exon 9/exon 11 | R419H/R550S | 11452359 | NaN | > 8 | NaN | NaN |
| 7 | Homozygous | exon 8 | R408X | 11452359 | NaN | > 8 | NaN | NaN |
| 8 | Homozygous | exon 9 | R389H | 11452359 | NaN | > 8 | NaN | NaN |
| 9 | Homozygous | exon 9 | R419P | 11452359 | NaN | > 8 | NaN | NaN |
| 10 | Compound heterozygous | exon 3/exon 9 | E197X/N437K | 11668628 | 432 | NaN | NaN | NaN |
| 11 | Compound heterozygous | exon 9/intron 12 | R419H/IVS12+1G>A | 11855938, 12777658 | 707.66 | NaN | NaN | NaN |
| 12 | Homozygous | exon 3 | del388-428/ins388-408 | 15375183 | 290.03 | 52.2 | NaN | NaN |
| 13 | Compound heterozygous | intron11/exon4 | IVS11+3insT/E146X | 16029460 | 193.35 | 57.67 | YES | YES |
| 14 | Compound heterozygous | intron11/exon4 | IVS11+3insT/E146X | 16029460 | 255.22 | 60.77 | YES | YES |
| 15 | Homozygous | exon 2 | E77X | 16029460, 17785700 | 197.22 | 38.04 | YES | YES |
| 16 | Homozygous | exon 2 | E77X | 16029460, 17785700 | 143.08 | 25.08 | YES | YES |
| 17 | Homozygous | exon 2 | E77X | 16029460, 17785700 | NaN | 16.16 | YES | YES |
| 18 | Compound heterozygous | exon 10/exon 11 | R446X/H510D | 17228349 | 224.67 | 14.6 | NaN | NaN |
| 19 | Homozygous | exon 10 | R446X | 17976197 | 382.06 | 15.76 | NaN | NaN |
| 20 | Homozygous | exon 10 | R446X | 17976197 | 246.33 | 12.77 | NaN | NaN |
| 21 | Homozygous | exon 10 | R446X | 17976197 | 200.08 | 13.93 | NaN | NaN |
| 22 | Homozygous | exon 7 | F283Ffs5X | 19111681 | 391.11 | 27.5 | NaN | NaN |
| 23 | Compound heterozygous | exon 7/exon 10 | F283Ffs5X/R446X | 19111681 | 416.24 | 21.4 | NaN | NaN |
| 24 | Compound heterozygous | exon 8/exon 9 | Y329X/N437K | 20521169 | 429.31 | NaN | NO | NO |
| 25 | Compound heterozygous | exon 9/exon 10 | R389H/R446X | 20521169 | 708.82 | 7.1 | NO | NO |
| 26 | Compound heterozygous | exon 9/exon 10 | R389H/R446X | 20521169 | 404.18 | 9.17 | NO | NO |
| 27 | Homozygous | exon 9 | R389H | 20521169 | 643.47 | 7.07 | NO | NO |
| 28 | Compound heterozygous | exon 9/exon 7 | R389H/G269R | 20521169 | 344.86 | 6.14 | NaN | NaN |
| 29 | Compound heterozygous | exon 1/exon10 | Q16X/R446X | 20719861 | 1028.54 | 8.4 ^a^ | NaN | NaN |
| 30 | Homozygous | exon 1 | Q22X | 24166850 | 205.72 | 27.6 | YES | YES |
| 31 | Homozygous | exon 1 | Q22X | 24166850 | 220.03 | 48.52 | YES | YES |
| 32 | Homozygous | exon 1 | Q22X | 24166850 | 134.57 | 35.98 | YES | YES |
| 33 | Homozygous | exon 10 | R446X | 24166850 | 220.03 | 57.54 | YES | YES |
| 34 | Compound heterozygous | Intron 9/exon10 | IVS9＋2A>G/R446X | 24166850 | 331.79 | 48.9 | YES | YES |
| 35 | Compound heterozygous | Intron 9/exon10 | IVS9＋2A>G/R446X | 24166850 | 143.85 | 29.64 | YES | YES |
| 36 | Compound heterozygous | Intron 9/exon10 | IVS9＋2A>G/R446X | 24166850 | 343.78 | 23.64 | YES | YES |
| 37 | Compound heterozygous | Intron 9/exon10 | IVS9＋2A>G/R446X | 24166850 | 338.75 | 61.93 | YES | YES |
| 38 | Homozygous | Exon 9 | R419H | 24166850 | 315.93 | 68.91 | YES | YES |
| 39 | Homozygous | Exon 7 | g.712G> A | 24166850 | 227.77 | 90.23 | YES | YES |
| 40 | Homozygous | intron10 | IVS10-1 G>T | 24623560 | NaN | 12.8 | YES | YES |
| 41 | Compound heterozygous | Exon 12/exon 9 | P558Efs14X/R389H | 25665839 | 525.83 | NaN | NaN | NaN |
| 42 | Compound heterozygous | Exon 12/exon 9 | P558Efs14X/R389H | 25665839 | NaN | NaN | NaN | NaN |
| 43 | Compound heterozygous | exon 1/exon 9 | S44A/R389H | 25665839 | 874.72 | NaN | NaN | NaN |
| 44 | Compound heterozygous | exon12/exon 10 | P558Efs14X/R446X | 25665839 | 760.10 | NaN | NaN | NaN |
| 45 | Homozygous | exon 10 | R446X | 26813946 | 300.00 | 20.20 | NaN | NaN |
| 46 | Heterozygote | intron 1/exon 11 | IVS1-1G>A/L511X | 26892138 | 295.00 | 68.10 | NaN | YES |
| 47 | Compound heterozygous | exon 9/intron 12 | R419H/IVS12-1G>A | 27170062 | 681.00 | 182.00 | NaN | NaN |
| 48 | Compound heterozygous | exon 9/exon 9 | R419H/R389H | 27401767, 28203044 | 866.00 | 24.60 | NO | NO |
| 49 | Compound heterozygous | exon 6/exon 10 | Q251X/R446X | 28521186 | 770.00 | 78.80 | NO | NO |

**^a^Sitosterolemia was initially ruled out because of the normal sitosterol:cholesterol ratio, but after one year sitosterol level increased to 8.4 mg/dL.**

**Supplementary Table 4.** Phenotype survey of the proband

| **Variables** | Pre-study | 6 Weeks | 9 Weeks | 14 Weeks | 18Weeks | 22 Weeks | 26Weeks | 34 Weeks | 68 Weeks | 75 Weeks | 127Weeks | 131Weeks | 136Weeks | 140 Weeks | **Normal Value of Rang** |
| --- | --- | --- | --- | --- | --- | --- | --- | --- | --- | --- | --- | --- | --- | --- | --- |
| **TC(mg/dl)** |  |  |  |  |  |  |  |  |  |  |  |  |  |  | <200 |
| Value | 638.06 | 491.11 | 398.30 | 266.44 | 286.16 | 221.97 | 247.49 | 324.83 | 286.16 | 294.28 | 193.35 | 222.74 | 154.29 | 141.53 |  |
| Change from prestudy |  | -146.95 | -239.75 | -371.62 | -351.90 | -416.09 | -390.57 | -313.23 | -351.90 | -343.78 | -444.71 | -415.32 | -483.76 | -496.52 |  |
| **LDL-C(mg/dl)** |  |  |  |  |  |  |  |  |  |  |  |  |  |  | <120 |
| Value | 527.46 | 406.04 | 343.39 | 187.94 | 208.04 | 156.61 | 182.91 | 243.62 | 172.85 | 157.00 | 109.82 | 136.12 | 71.54 | 51.43 |  |
| Change from prestudy |  | -121.42 | -184.07 | -339.52 | -319.41 | -370.85 | -344.55 | -283.84 | -354.60 | -370.46 | -417.64 | -391.34 | -455.92 | -476.03 |  |
| **LDH(Iu/l)** | 225.70 | 201.70 | 182.20 | 206.20 | 199.40 | 200.10 | 189.70 | 185.70 | 154.90 | 228.80 | 176.60 | NaN | 183.40 | 204.70 | 80~190 |
| **CK-MB(Iu/l)** | 21.00 | 20.40 | 22.70 | 33.00 | 21.00 | 26.74 | 24.90 | 17.60 | 20.80 | 26.70 | 22.30 | NaN | 18.70 | 22.30 | 0~25 |
| **GPT(u/l)** | 20.20 | 34.20 | 5.10 | 6.45 | 5.05 | 5.29 | 5.30 | 5.50 | 3.60 | 6.70 | 5.30 | NaN | 5.00 | 5.70 | 5~40 |
| **AST(u/l)** | 41.50 | 56.00 | 27.50 | 29.67 | 29.73 | 28.16 | 28.20 | 31.30 | 25.70 | 29.70 | 23.70 | NaN | 27.60 | 27.30 | 5~40 |
| **ALP(u/l)** | 240.90 | 243.80 | 215.70 | 216.80 | 217.80 | 222.70 | 216.60 | 200.50 | 201.10 | NaN | 270.50 | NaN | 257.50 | 256.10 | 34~104 |

Lactate dehydrogenase, LDH; Creatinine kinase, MB isoenzyme, CK-MB; Glutamic-pyruvic transaminase, GPT; Aspartate transaminase, AST; Alkaline phosphatase, ALP.

**Supplementary Table 5.** Nine variations identified in 30 individuals with abnormal platelet distribution

| **hg19** | **Variant_ID** | **REF** | **ALT** | **Consequence** | **alt allele count** | **all allele counts** | **alt frequency** | **#homozygotes** | **Xinjian Cohort (patient ID)** |
| --- | --- | --- | --- | --- | --- | --- | --- | --- | --- |
| **chr2:44039633** | **rs4148195** | **G** | **A** | **c.*622C>T | 3’UTR** | **36488** | **151740** | **0.24** | **5308** | **2,5,8,9,16,18,27** |
| **chr2:44039644** | **v3** | **T** | **TTAATCCTCAGT**  **AAAATCACTGGG** |  | **-** | **-** | **-** | **-** | **23** |
| **chr2:44039650** | **v4** | **C** | **CTCAGTAAAATC**  **ACTGGGTGCTCTGTA** |  | **-** | **-** | **-** | **-** | **14** |
| **chr2:44039839** | **rs2278357** | **C** | **T** | **c.*416G>A | 3’UTR** | **33312** | **152020** | **0.219** | **4266** | **2,4,18,27** |
| **chr2:44039875** | **rs2278356** | **A** | **C** | **c.*380T>G | 3’UTR** | **66283** | **151930** | **0.436** | **16244** | **4,5,8,9,16,18,27** |
| **chr2:44040401** | **rs6720173** | **G** | **C** | **p.Gln604Glu | missense** | **32178** | **152082** | **0.212** | **3874** | **18** |
| **chr2:44050063** | **rs199689137** | **G** | **A** | **p.Arg446Ter | stop-gain** | **19** | **152042** | **0.000125** | **0** | **30** |
| **chr2:44065090** | **rs6756629** | **G** | **A** | **p.Arg50Cys | missense** | **10274** | **152086** | **0.0676** | **425** | **2,4,10,11,16,27** |
| **chr2:44065755** | **rs781098379** | **G** | **A** | **p.Gln22Ter | stop-gain** | **-** | **-** | **-** | **-** | **28** |

**Alternative allele count, all allele counts, alternative frequency and number of homozygotes are all from gnomAD database.**

**IV. Supplementary References**

1. Kasama T, Byun DS, Seyama Y. Quantitative analysis of sterols in serum by high-performance liquid chromatography. Application to the biochemical diagnosis of cerebrotendinous xanthomatosis. J Chromatogr 1987;400:241-6.
2. Hidaka H, Nakamura T, Aoki T, et al. Increased plasma plant sterol levels in heterozygotes with sitosterolemia and xanthomatosis. J Lipid Res 1990;31:881-8.
3. Barsam SJ, Psaila B, Forestier M, et al. Platelet production and platelet destruction: assessing mechanisms of treatment effect in immune thrombocytopenia. Blood 2011;117:5723-32.
4. Depoorter M, Goletti S, Latinne D, Defour J. Optimal flagging combinations for best performance of five blood cell analyzers. Int J Lab Hematol 2015;37:63-70.
5. Martin M: Cutadapt removes adapter sequences from high-throughput sequencing reads. 2011 2011, 17(1).
6. Li H, Durbin R: Fast and accurate short read alignment with Burrows-Wheeler transform. Bioinformatics 2009, 25(14):1754-1760.
7. McKenna A, Hanna M, Banks E, Sivachenko A, Cibulskis K, Kernytsky A, Garimella K, Altshuler D, Gabriel S, Daly M et al: The Genome Analysis Toolkit: a MapReduce framework for analyzing next-generation DNA sequencing data. Genome research 2010, 20(9):1297-1303.
